# Role of miRNA dysregulation in sepsis

**DOI:** 10.1186/s10020-022-00527-z

**Published:** 2022-08-19

**Authors:** Amanda Formosa, Paul Turgeon, Claudia C. dos Santos

**Affiliations:** 1 Interdepartmental Division of Critical Care Medicine, University of Toronto, Toronto, Canada; 2 The Keenan Research Centre for Biomedical Science, St. Michael’s Hospital, Unity Health Toronto, 209 Victoria Street, Toronto, ON M5B1T8 Canada; 3 Department of Laboratory Medicine and Pathobiology, University of Toronto, Toronto, ON M5S 1A8 Canada; 4 Institute of Medical Sciences, Temerty Faculty of Medicine, University of Toronto, 1 King’s College Circle, Toronto, ON M5S 1A8 Canada; 5 Department of Physiology, Faculty of Medicine, University of Toronto, 1 King’s College Circle, Toronto, ON M5S 1A8 Canada

**Keywords:** miRNAs, Small RNAs, Sepsis, Septic shock, Endothelium, Extracellular vesicles, Myeloid-derived suppressor cells, Reference gene

## Abstract

**Background:**

Sepsis is defined as a state of multisystem organ dysfunction secondary to a dysregulated host response to infection and causes millions of deaths worldwide annually. Novel ways to counteract this disease are needed and such tools may be heralded by a detailed understanding of its molecular pathogenesis. MiRNAs are small RNA molecules that target mRNAs to inhibit or degrade their translation and have important roles in several disease processes including sepsis.

**Main body:**

The current review adopted a strategic approach to analyzing the widespread literature on the topic of miRNAs and sepsis. A pubmed search of “miRNA or microRNA or small RNA and sepsis not review” up to and including January 2021 led to 1140 manuscripts which were reviewed. Two hundred and thirty-three relevant papers were scrutinized for their content and important themes on the topic were identified and subsequently discussed, including an in-depth look at deregulated miRNAs in sepsis in peripheral blood, myeloid derived suppressor cells and extracellular vesicles.

**Conclusion:**

Our analysis yielded important observations. Certain miRNAs, namely miR-150 and miR-146a, have consistent directional changes in peripheral blood of septic patients across numerous studies with strong data supporting a role in sepsis pathogenesis. Furthermore, a large body of literature show miRNA signatures of clinical relevance, and lastly, many miRNAs deregulated in sepsis are associated with the process of endothelial dysfunction. This review offers a widespread, up-to-date and detailed discussion of the role of miRNAs in sepsis and is meant to stimulate further work in the field due to the potential of these small miRNAs in prompt diagnostics, prognostication and therapeutic agency.

**Supplementary Information:**

The online version contains supplementary material available at 10.1186/s10020-022-00527-z.

## Background

Sepsis is defined as a dysregulated host immune response to infection that leads to multisystem organ damage (Singer et al. 2016). The true incidence of sepsis in any given country is unknown due to differences in sepsis definitions, reporting mechanisms and the origin of data on sepsis, which is usually from high income countries (Cecconi et al. 2018). One can certainly estimate that the disease claims the life of millions of people around the world annually and costs billions of dollars to health care systems (Cecconi et al. 2018; Angus et al. 2001). Due to the variable clinical presentations of sepsis, rapid diagnosis can prove challenging. Prompt recognition and treatment improve patient outcome, since every one-hour delay in starting antimicrobial therapy increases mortality by 7.6% (Kumar et al. 2006; Evans et al. 2021). Research efforts on novel treatments of sepsis in clinical trials have had disappointing results, partially owing to a lack of understanding into the disease’s pathophysiology (Ranieri et al. 2012; Giza et al. 2016). Biomedical research into the pathogenesis of sepsis holds promise for pathway-specific drug discovery to revolutionize the course of this illness.

Sepsis has a complex pathophysiology with features of pro-inflammation, immune suppression and endothelial dysfunction. A firmly established pathway to immune activation occurs when the host generates a proinflammatory reaction against the invading pathogen (Angus and Poll 2013). Cells of the innate immune system [neutrophils, monocytes, macrophages) herald the initial inflammatory response by interacting with pathogens via pathogen recognition receptors [PRRs), an example of which is the toll like receptor class (TLRs) (Boyd et al. 2014). These receptor classes recognize conserved structures among microbes called pathogen-associated molecular patterns (PAMPs), such as the lipopolysaccharide (LPS) component of the outer membrane of gram-negative bacteria, to initiate NF-κB/RelA/p65 transcription and mitogen activated protein kinases (Brudecki et al. 2013). The resulting systemic inflammatory response increases levels of proinflammatory cytokines including tumor necrosis factor-alpha (TNF-α), IL6, IL8 and IL-1b (Giza et al. 2016; Brudecki et al. 2013).

The proinflammatory cascade in sepsis, while aimed at the invading pathogen, causes collateral tissue damage (Angus and Poll 2013). To avoid this detrimental effect, the immune system adapts an anti-inflammatory and immunosuppressive profile referred to as endotoxin tolerance/adaptation. The gold standard definition of endotoxin tolerance is decreased release and circulation of proinflammatory molecules in response to TLR4’s repeat exposure to LPS or bacterial endotoxin (Cavaillon and Adib-Conquy 2006). Markers such as IL4, IL10, IL13, IkBα and IL1Ra indicate a more anti-inflammatory milieu (Giza et al. 2016; Brudecki et al. 2013). Immunosuppression makes the host more susceptible to secondary infections.

Sepsis is characterized by an excessive, sustained and generalized activation of the endothelium, clinically presenting as increased thrombosis, tissue inflammation and edema (Ince et al. 2016; Joffre et al. 2020). Endothelial cells (ECs) take on a proinflammatory phenotype when they interact with PRRs, releasing cytokines and pro-coagulation molecules. The interstitial leakage in sepsis derives from the increased permeability associated with damage to the glycocalyx and EC apoptosis (Colbert and Schmidt 2016). The changes in ECs with sepsis are partly beneficial to limit bacterial spread, but persistent endothelial changes lead to poor microcirculatory blood flow and tissue hypoperfusion (Joffre et al. 2020). Teasing apart an appropriate endothelial response from a maladaptive one is challenging and complicated by the fact that the endothelium is a structure with gross heterogeneity.

In the past fifteen years, microRNAs (miRNAs) have emerged as key players in sepsis pathogenesis. MiRNAs are endogenous non-coding RNA molecules of approximately 21 nucleotides in length (Bartel 2004). In 1993, Lee and colleagues discovered the first miRNA, lin-4 in *C. elegans*, and elucidated its role in development (Lee et al. 1993). MiRNAs affect gene expression by binding to an mRNA’s 3’-untranslated region (UTR) to degrade or inhibit protein translation (Bartel 2004). Since their discovery, miRNAs have been implicated as key players across a myriad of cellular and organismal functions, such as development, differentiation, cell death and senescence (Formosa et al. 2014; Ivey and Srivastava 2015; Zhang et al. 2017). Importantly, they have emerged as putative biomarkers for many diseases and realistic targets for therapeutic strategies that have reached clinical trials (Rupaimoole and Slack 2017). For example, in an open label, dose escalation, multicentre phase I trial in Australia, an acceptable safety profile was established for TargomiRs (miRNA loaded minicells) in malignant mesothelioma (Zandwijk et al. 2017). Understanding the role of miRNAs in sepsis lends to a unique and plausible angle of discovery for novel ways to diagnose, prognosticate and treat this disease.

The current review aims to provide a comprehensive analysis on the current landscape of miRNAs in human sepsis, with an emphasis on what is known about miRNA deregulation in human peripheral blood. In addition, we sought to understand the mechanisms of action of deregulated miRNAs in sepsis, and thus included many articles on miRNA function in mouse or in vitro models of sepsis. To ensure that this work offered the most comprehensive understanding of the literature on the topic, we adopted a methodical approach to gathering studies of interest, as outlined below.

## Approach to current review

This review summarizes relevant manuscripts available on PubMed up to and including January 2021 as a result of the search terms, “miRNA or microRNA or small RNA and sepsis not review”. The search resulted in 1140 abstracts. To avoid predatory journals, only those listed on the Committee on Publication Ethics website (COPE, https://publicationethics.org/) and/or with an impact factor greater than or equal to 2 were included. A total of 907 paper were eliminated, mainly due to irrelevance. The first relevant paper was in 2007 by Tili et al. on miR-155 and miR-125b levels after LPS stimulation of mouse macrophages (Tili et al. 2007). The first report on a human miRNAome profiling in the blood of sepsis patients was published in 2009 by Vasilescu et al. (2009). At the end of our search strategy, 233 original research papers were included and meticulously analyzed for important discoveries and recurring themes, which are discussed below.

## Deregulation of circulating miRNAs in human peripheral blood

Additional file 1: Table S1 summarises the numerous studies that have elucidated differentially expressed miRNAs in the peripheral blood of human subjects with sepsis. The scientific goal in this context has been (1) to unravel miRNAs with diagnostic and prognostic potential given the ease of accessibility of peripheral blood to testing and (2) understand the mechanism behind specific miRNA deregulation in peripheral blood so as to offer new avenues to pathogenesis elucidation.

### Technical considerations

A proper understanding of miRNA deregulation in peripheral blood necessitates an appreciation for nuances about the methodological approach. Additional file 1: Table S1 records key details for each study that we analyzed. We noted the precise patient population and its size: pediatric versus adult; sepsis versus septic shock; sepsis survivors versus non survivors; volunteers as healthy controls (HCs) versus control patients in the ICU without sepsis. One should note the component of peripheral blood from which miRNAs were extracted (for example, whole blood versus T cells) as this may affect the expression profile in a disease-specific manner (Reithmair et al. 2017; Mohnle et al. 2018).

The role of the internal reference control for quantitative reverse transcription polymerase chain reaction (RT-qPCR) stands as an essential methodological feature to appreciate. For every study included in our search, the reference control was documented in Additional file 1: Table S1. Benz et al. have documented the inappropriateness of the commonly used U6 small RNA as a reference standard for investigating miRNA deregulation in the sera of human and mice with sepsis using RT-qPCR (Benz et al. 2013). Strikingly, U6 exhibited disease specific alterations in critical illness and sepsis compared to controls. U6 was also found to be poorly stable in the blood and circulating T cells of septic patients and HCs (Hirschberger et al. 2019). On the other hand, spiked in SV-40 had better stability across groups. Spike in controls serve to normalize across extractions but does not yield any insight into systemic changes. A better alternative for normalization proposed by Schlosser et al. involved screening hundreds of miRNAs for their stability in the clinical context at hand (Schlosser et al. 2015). Their normalization method echoes the schematics used by software programs like Genorm and Normfinder, which many authors have incorporated in their analysis as shown in Additional file 1: Table S1 (Hellemans and Vandesompele 2014).

Further technological aspects to consider include the content of hemolyzed RBCs in blood (may bias miRNA levels in plasma) (Pritchard et al. 2012; Kirschner et al. 2011), which few authors have taken into account in their methodologies (Caserta et al. 2016). Other contributors to variability in results across studies may include the type and source of sepsis (bacterial gram negative or positive versus viral in origin) (How et al. 2015; Yousefpouran et al. 2020).

With a good foundation on how to interpret the studies listed in Additional file 1: Table S1, one can appreciate which miRNAs have convincing changes in human peripheral blood during sepsis. Most miRNAs deregulated in peripheral blood are only noted in one to two or three reports. Caserta et al. nicely articulated the clear lack of unanimous results across studies with regards to which miRNAs are deregulated in peripheral blood of septic patients and Additional file 1: Table S1 certainly supports that interpretation (Caserta et al. 2016).

To bring forth miRNAs showing consistent deregulation in septic patients’ peripheral blood across studies, each miRNA noted in Additional file 1: Table S1 was cross referenced against all studies listed within that same table. Table 1 lists miRNAs noted in more than one study along with the direction of change in peripheral blood of septic patients. Given that miR-150 and miR-146a had remarkably consistent findings, they will be discussed in more detail next.

**Table 1:** Direction of fold change of miRNAs in human peripheral blood that are implicated in sepsis (in order of increasing miRNA)

miRNA	Direction of change	References
miR-1-3p	↑	Gao et al. (2021)
miR-15a	↑	Mohnle et al. (2018)
	↑ (in sepsis compared to HCs); ↓ in septic shock compared to sepsis	Goodwin et al. (2015)
	↑	Wang et al. (2012d)
	↑ (in non-survivors)	Wang et al. (2012b)
miR-16	↑ in septic T cells; ↓ in whole blood (separate cohorts)	Mohnle et al. (2018)
	↑	Goodwin et al. (2015)
	↓ in FCH cohort and ↑ in MDACC cohort	Tudor et al. (2014)
	↔ between SIRS and sepsis; ↑ sepsis to HCs	Wang et al. (2012d)
	↓ in non-survivors	Wang et al. (2012b)
miR-21	↑	Zhang et al. (2019)
	↑	Xue et al. (2019)
	↑	Sheng et al. (2017)
	↑	Goodwin et al. (2015)
miR-23b	↑ in early onset sepsis	Fatmi et al. (2020)
	↓ in late sepsis and death	
miR-29b-3p	↓	Li et al. (2020b)
miR-96-5p	↓	Chen et al. (2020a)
miR-103a-3p (synonymous with miR-103)	↑	Zhou and Xia 2020)
	↓	Yang et al. (2020a)
	↓	Wang et al. (2020a)
miR-106a	↑	Heng et al. (2020)
miR-107	↓	Wang et al. (2020a)
miR-122	↔	Roderburg et al. (2019)
	↑	Rahmel et al. (2018)
	↑	Real et al. (2018)
	↓	Wang et al. (2014)
	↓ (severe sepsis/septic shock)	Wang et al. (2012a)
	↑ in non-survivors	Wang et al. (2012b)
miR-125a	↔	Zhu (2020)
	↑	Li et al. (2020c)
	↓	Liu et al. (2020a)
	↓	Yang et al. (2020b)
miR-125b	↑	Zhu (2020)
	↑	Li et al. (2020c)
miR-126	↑	Goodwin et al. (2015)
	↓	Wang et al. (2010)
	↑	Lin et al. (2020)
miR-128-3p	↓	Yang et al. (2020c)
miR-133a	↑	Roderburg et al. (2019)
	↑	Tacke et al. (2014)
	↑	Chen et al. (2020c)
miR-142-5p	↓	Qin et al. (2020)
	↓	Zhen and Chen (2018)
	↓	Ge et al. (2017b)]
miR-143	↑	Qin et al. (2020)
	↓ (in non-survivors)	Roderburg et al. (2019)
	↑	Mohnle et al. (2018)
	↑	Han et al. (2016)
	↑	Zhou et al. (2015)
	↑	Schmidt et al. (2009)
miR-145	↓	Cao et al. (2019)
	↑	Zhang et al. (2019)
	↑	Zhou et al. (2015)
miR-146a	↓	Karam et al. (2019)
	↑	Goodwin et al. (2015)
	↓	Mohnle et al. (2015)
	↓	Zhou et al. (2015)
	↓	Tudor et al. (2014)
	↓	Shao et al. (2014)
	↓	Wang et al. (2013a)
	↓	Wang et al. (2010)
	↑	Chen et al. (2020d)
miR146b	↑	Chen et al. (2020d)
	↓	Chen et al. (2020b)
miR-150	↓ (in non-survivors compared to survivors)	Braza-Boils et al. (2020)
	↓	Tacke et al. (2014)
	↓	Ma et al. (2018)
	↓	Mohnle et al. (2018)
	↓	How et al. (2015)
	↓	Tudor et al. (2014)
	↑	Ma et al. (2013)
	↔ serum levels; ↓ serum predicted mortality	Roderburg et al. (2013)
	↓	Schmidt et al. (2009)
	↓	Vasilescu et al. (2009)
miR-155	↔	Tacke et al. (2019)
	↔	Roderburg et al. (2019)
	↑	Vasques-Novoa et al. (2018)
	↑	Liu et al. (2015)
	↓	Zhou et al. (2015)
	↔	Wang et al. (2010)
	↔	Goodwin et al. (2015)
miR-181a-5p (synonymous with miR-181a)	↓	Liu et al. (2020b)
	↓	Wang et al. (2020b)
miR-182	↑	Zhou et al. (2015)
	↑	Tudor et al. (2014)
	↑	Vasilescu et al. (2009)
miR-19b-3p	↓	Xu et al. (2020)
miR-191	↓	Ge et al. (2017b)
	↓	Caserta et al. (2016)
	↑	Ma et al. (2013)
miR-21	↓	Na et al. (2020)
miR-204-5p	↓	Chen and Song (2020)
miR-206	↑	Liang et al. (2020)
miR-223	↑	Qin et al. (2020)
	↑	Zhang et al. (2019)
	↓	Roderburg et al. (2019)
	↑	Mohnle et al. (2018)
	↑	Wu et al. (2018)
	↔	Benz et al. (2015)
	↑	Goodwin et al. (2015)
	↑ in sepsis compared to NCs; ↓ in septic shock compared to sepsis	Wang et al. (2012a)
	↓	Wang et al. (2012b)
	↓	Wang et al. (2010)
	↑ in sepsis compared to NCs; ↓ in non-survivors compared to survivors	Liu et al. (2020c)
miR-328	↑	Liu et al. (2020c)
miR-342-3p	↓	Mohnle et al. (2018)
	↓ septic MNCs; ↓	Tudor et al. (2014)
	FHC cohort; ↑	
	MDACC cohort	
	↑	Ma et al. (2013)
	↓	Schmidt et al. (2009)
miR-452	↑	Liu et al. (2020d)
miR-483-5p	↓	Wang et al. (2014)
	↑ (sepsis vs. HCs); ↓	Wang et al. (2012a)
	(severe sepsis/shock vs. sepsis)	
	↑ (in non-survivors)	Wang et al. (2012b)
miR-486	↑	Goodwin et al. (2015)
	↑	Zhou et al. (2015)
	↑	Vasilescu et al. (2009)
miR-545	↑	Wei and Yu (2020)
miR-590-3p	↓	Liu et al. (2020e)
miR-1246	↓	Hermann et al. (2020)

↑ is upregulated in sepsis, ↓ is downregulated in sepsis and ↔ is no significant difference. Important features of study cohort are noted

### Circulating miR-150 in sepsis

Table 1 shows that miR-150 had differential expression in sepsis in nine studies, eight of which exhibited downregulation (Vasilescu et al. 2009; Mohnle et al. 2018; How et al. 2015; Tacke et al. 2014; Ma et al. 2018, 2013; Tudor et al. 2014; Roderburg et al. 2013; Schmidt et al. 2009). We reviewed the miR-150 literature for mechanisms to explain the consistent downregulated trend.

Ma et al. found that miR-150 plasma levels in human patients with sepsis had diagnostic and prognostic value, correlating negatively with renal dysfunction, plasma levels of interleukin-6 (IL-6), TNF-α and 28-day survival (Ma et al. 2018). The authors used human umbilical vein endothelial cells (HUVECs) and septic C57BL/6 mice after lipopolysaccharides (LPS) treatment as models to understand the miR-150’s mechanistic action. Overexpression of miR-150 protected HUVECs from LPS-induced apoptosis. MiR-150 was found to directly target NF-κB1. The authors concluded that miR-150 predicted survival in patients with sepsis, possibly by exerting an anti-inflammatory and anti-apoptotic role by targeting NF-κB1.

In another study focused on vascular leakage and sepsis, Rajput et al. investigated the role of miR-150 and Angiopoietin (Ang2) in a miR-150^−/−^ mouse model (Rajput et al. 2016). Ang2 induces vascular leakage and correlates with patient mortality in sepsis. Since miR-150 was shown to be decreased in sepsis, the authors hypothesized that miR-150 suppresses Ang2 generation to ameliorate vascular injury. Wild-type or *miR-150*^*−/−*^ mice or endothelial cells exposed to LPS or sepsis were assessed for Ang2 levels, adherens junction re-annealing, endothelial barrier function and mortality. Ang2 transiently increased during LPS-induced injury in wild-type endothelial cells and mouse lungs. MiR-150 expression was elevated during the injury recovery phase. Deletion of miR-150 caused a persistent increase in Ang2 levels and impaired adherens junctions re-annealing after injury, which led to increases in vascular permeability. M*iR-150*^*−/−*^ mice died rapidly after sepsis. Rescuing miR-150 expression in endothelial cells prevented Ang2 generation and restored vascular barrier function in *miR-150*^*−/−*^ mice. Early growth response 2 or Ang2 depletion in *miR-150*^*−/−*^ endothelial cells restored barrier function. Re-expression of miR-150 by injecting a chemically synthesized miR-150 mimic improved sepsis mortality.

MiR-150 has been demonstrated to have a role in immune modulation. A possible role for miR-150 in septic T cell immunoparalysis was underlined by Mohnle et al. (2018). The authors assessed for differentially regulated miRNAs in purified T-cells or whole blood cells obtained from septic patients and healthy volunteers. T-cells of septic patients had immunosuppressive characteristics in that several pro-inflammatory molecules such as miR-150 and miR-342 were downregulated, while a host of anti-inflammatory like miR-15a and miR-16 were upregulated. MiR-143, miR-150 and miR-223 levels indicated T-cell immunoparalysis and correlated with patient SOFA-scores. MiR-143 and miR-150 (both predominantly expressed in T-cells) retained strong power of discrimination in whole blood. The authors concluded that miR-143 and miR-150 are promising markers for T-cell immunosuppression in whole blood.

MiR-150’s role in sepsis has also been illustrated in studies of its target genes. Schmidt et al. noted multiple predicted targets at the computational level for miR-150 in pathways key to the sepsis process, including innate pathogen detection (IRAK2, MAP2K4), apoptosis signalling (BBC3 and BCL2L2), cytokine signalling (PDGFRA) and MAPK/NF-κB signaling (AKT3 and EIF4E) (Schmidt et al. 2009). Vasilescu et al. also used computational programs to see whether miR-150 target genes had a role in sepsis pathogenesis and several sepsis-related pathways emerged: MAPK, insulin resistance, Wnt, ErbB and mTOR. (Vasilescu et al. 2009). MiR-150 has also been shown to target the chemokine receptor type 4 (CXCR4), which plays an important role in stem cell mobilization and migration in ischemic tissues (Tano et al. 2011). Ischemia inhibits the expression of miR-150 in bone marrow-derived mononuclear cells and this consequently activates the CXCR4 target gene.

Our literature review on miR-150 revealed its involvement in vascular leakage, immune modulation and inflammation. Together with the notion that its expression has a consistent pattern in human peripheral blood across studies, this miRNA has a convincing role in sepsis pathogenesis.

### miR-146a and sepsis

Our search on deregulated miRNAs in sepsis resulted in seven studies with significant and consistent downregulation of miR-146a in peripheral blood. Karam et al. examined circulating miR-146a levels in septic children compared to age and gender matched HCs (Karam et al. 2019). The serum levels of miR-146a were significantly decreased in sepsis, with an even stronger signal in septic shock and correlated with survival.

The remaining six studies had similar results for miR-146a in sepsis despite technical differences in study design, such as varying sample sizes and type of control group. Tudor et al. performed miRNAomic microarray analysis of mononuclear cells (primarily leukocytes) from eight septic and eight healthy humans and showed a downregulation for miR-146a in sepsis (Tudor et al. 2014). Other studies looking at miR-146a in PBMCs or T cells with HCs (HCs) as a baseline showed the same downregulated trend (Zhou et al. 2015; Shao et al. 2014; Mohnle et al. 2015). Again, the same trend of decreased miR-146a levels was noted by Wang et al. specifically in the plasma of 4 sepsis patients (Wang et al. 2013a). Interestingly, the control group used here was 6 non-sepsis SIRS, leading one to postulate that lower levels of miR-146a may very well be attributed to sepsis rather than systemic inflammation. In another study that included non-septic SIRS as a control group, miR-146a was significantly lower in the sera of SIRS patients (n = 30) compared to HCs (n = 20), but was more downregulated in sepsis patients (n = 50) compared to SIRS (Wang et al. 2010).

As reviewed by Testa et al. , miR-146a has emerged as an important regulator of both innate and adaptive immunity, with key expression and function in various cell types of the immune system, including dendritic, CD8 + T lymphocyte, Tfh, Th1, Thr and Th 17 cells (Testa et al. 2017). In monocytes and macrophages for example, miR-146a expression increases after LPS stimulation via the NF-κB pathway and causes a decrease in inflammatory cytokines. MiR-146a has been shown to be affected by various inflammatory stimuli including some cytokines and TLRs in various cell types (Labbaye and Testa 2012).

MiR-146a targets several TLR4 effectors, including TNFR-associated factor 6 (TRAF6), IRAK1, IRAK2, IRF3 and IRF5. MiR-146a has also been shown to positively affect endotoxin tolerance (Nahid et al. 2009; Doxaki et al. 2015). In a report by Nahid et al. , gradual increases in miR-146a were observed in THP-1 cells at 4 h after LPS stimulation, continuing up to 35-fold over 24 h with a converse decrease in TNF-α expression. MiR-146a overexpression was observed in tolerized THP-1 cells. Transfection of miR-146a into THP-1 cells was similar to LPS priming, whereas miR-146a inhibition significantly decreased LPS tolerance, thus illustrating miR-146a’s critical role in an in vitro model of monocytic cell-based endotoxin tolerance.

In addition to cells of the immune system, miR-146a’s mechanism of action has been illustrated in the endothelium. In a study by Gao et al. , miR-146a expression was significantly decreased in LPS-induced endothelial cells, and its suppression with an inhibitor increased NF-κB activity along with higher levels of IL-6, TNF-α, ICAM-1, and E-selectin (Gao and Dong 2017). In another in vitro sepsis model of the activated endothelium, miR-146a demonstrated anti-inflammatory properties by downregulating IL-8 and IL-6, and regulating the expression of heat shock protein-10 (HSP10) (Pfeiffer et al. 2017).

The molecular function of miR-146a has been illustrated in in vivo models of miR-146a (miR-146a −/−) deficient mice. As a negative feedback regulator of NF-κB signaling, mice that lack miR-146a display exaggerated inflammatory responses to bacterial exposure and develop a systemic autoimmune disease over time (Boldin et al. 2011; Zhao et al. 2011). In a recent study by Su et al. , synthetic C-miR146a mimic decreased NF-κB activity in macrophages and myeloid leukemia in vitro and in vivo, thus alleviating monocyte-mediated cytokine storm and inhibiting inflammation and tumorigenicity (Su et al. 2020). One striking feature of this report is the illustration of a feasible method for systemic delivery of miRNA mimic specifically to human and mouse myeloid cells for therapeutic modulation of immune activity or neoplastic growth. MiR-146a’s consistent downregulated trend in human peripheral blood, its role as an anti-inflammatory molecule and the plausibility of delivering it as a miRNA mimic highlights this miRNA as potential novel tool in the realm of treatment strategies.

### Clinically relevant miRNA signatures in peripheral blood

Several studies that emerged in our search evaluated miRNA signatures for their correlation to clinically relevant parameters, namely sepsis diagnosis, phases of sepsis and prognosis.

Shen et al. investigated the accuracy of circulating miRNAs in diagnosing sepsis from SIRS and HCs via a systematic review and meta-analysis (Shen et al. 2020). They included 22 records which had 2210 sepsis patients, 426 systemic inflammatory response syndrome (SIRS), and 1076 HCs. The pooled sensitivity and specificity of miRNAs were 0.80 and 0.85, respectively. Subgroup analysis indicated that improved diagnostic accuracy was achieved with miRNAs in adults, serum rather than plasma, downregulation of miRNA expression, criteria of Sepsis-3 versus sepsis-1 or sepsis-2, non-U6 internal reference for normalization, and dysregulated miR-223 expression. The search strategy adopted here also resulted in several reports identifying miR-223 expression as an important miRNA biomarker for sepsis (Ma et al. 2013; Wang et al. 2010, 2012a; Wu et al. 2018; Huang et al. 2014).

In a study by Ma et al. aiming to identify novel biomarkers for rapid sepsis diagnosis in the ICU, small RNA sequencing of whole blood was used to identify differentially expressed miRNAs in septic patients and controls (Ma et al. 2013). Using a linear discriminant statistical model, a composite signature of miR-150 and miR-4772-5p-iso was generated that possessed 90.5% specificity and 81.8% sensitivity in distinguishing sepsis from SIRS. The findings were then validated in an independent cohort. The results from the two cohorts showed an 86% diagnostic accuracy for sepsis. To investigate a mechanistic role for miR-4772-5p-iso in sepsis, in vitro studies were carried out that revealed this miRNA to be upregulated in primary peripheral blood monocytes after a 24 h challenge with specific TLR ligands. MiR-150 has been delineated as a sepsis biomarker elsewhere (Vasilescu et al. 2009).

Current literature suggested a vital role for miRNAs in distinguishing various stages of sepsis (Karam et al. 2019; Wu et al. 2018; Wang et al. 2012a; Liu et al. 2015). In a study by Wang et al. , 166 patients with sepsis and 24 normal controls were analyzed for serum expression levels of miR-223, miR-15b, miR-483-5p, miR-499-5p, miR-122, and miR-193b* by RT-qPCR assays. MiR-223 and miR-499-5p levels were significantly different in patients with mild sepsis, severe sepsis and septic shock. In a binary logistic regression model, only miR-499-5p and SOFA were able to distinguish between mild sepsis and severe sepsis and septic shock (Wang et al. 2012a). MiR-146a was implicated in stages of sepsis in a pediatric cohort, with lowest levels seen in septic shock (Karam et al. 2019). Similarly, miR-223 and miR-155 levels were shown to positively correlate with sepsis severity (Wu et al. 2018; Liu et al. 2015).

Circulating miRNAs have also been implicated as prognostic markers. In a study by Goodwin et al. , plasma samples from patients with severe sepsis (n = 62) and HCs (n = 32) were analyzed by RT-qPCR for candidate miRNAs (Goodwin et al. 2015). In the group who developed shock, miR-34a expression was significantly increased while miR-15a and miR-27a expressions were decreased in this group. The combined expression of these three miRNAs predicted shock with an area under the curve of 0.78. In silico analyses predicted that these three miRNAs regulate genes involved in endothelial cell cycle, apoptosis, VEGF signaling, LPS-stimulated MAPK signaling, and NF-κB signaling. Patient survival has also been predicted with relative accuracy using circulating miRNA profiles. In a cohort of 214 sepsis patients, a combination of 4 serum microRNAs (miR-15a, miR-16, miR-193* and miR-483-5p) and sepsis clinical scores predicted 28 day survival with a sensitivity of 88.5% and a specificity of 90.4% (Wang et al. 2012b). Many other miRNAs have been implicated in prognosis and/or survival, including miR-122, miR-150, miR-155, miR-193b*, miR-223, miR-483-5p, miR-574-5p and others (Vasilescu et al. 2009; Ma et al. 2018; Roderburg et al. 2013; Wu et al. 2018; Wang et al. 2012b, 2013b, 2012c; Tacke et al. 2019; Rahmel et al. 2018). Altogether, these results indicate circulating miRNAs could have the ability to screen and monitor the progress of sepsis.

### miRNAs and the endothelium in sepsis

Given the crucial role of a dysfunctional endothelium in sepsis, it is with no surprise that our search on miRNAs and sepsis yielded a significant number of reports focusing on blood vascular ECs. The first thing that stands out is miR-126, noted in three papers (Guo et al. 2016; Chu et al. 2016; Jones Buie et al. 2019). One of the earlier of the three papers found increased miR-126 aided in barrier function in cell culture (Guo et al. 2016). Chu et al. (2016) recapitulated results whereby decreased levels of miR-126-3p were detrimental to vascular permeability and proliferation and can alter adhesion proteins in mice aortas (Chu et al. 2016). Taking it one step further, delivery of miR-126-5p in mice improved survival (Jones Buie et al. 2019). A second notable sepsis miRNA in the endothelial cell was miR-150, which was discussed above..

Several papers have tried to address alterations in the function of endothelial cells with respect to miRNA deregulation and correlation with sepsis outcomes. One of the earlier papers compared between septic patients and human HCs found upregulated levels of miRNA-34a in patients who develop septic shock, while miR-15a and miR-27a were decreased (Goodwin et al. 2015). Interestingly, these miRNAs are believed to be relevant to particular endothelial responses in sepsis, such as LPS-stimulated signaling cascades, vascular endothelial growth factor (VEGF) signaling, and apoptosis. Of these three miRNAs, miR-34a was further interrogated in endothelial cells. It was shown that increasing miR-34a levels attenuated TNF-α and NF-κB activation while IL-10 and Notch-1 levels increased after LPS treatment (Ge et al. 2017a). Overall, the concurrent regulation of these pathways may reduce endothelial damage in vivo, but this remains to be tested.

An exceptional level of inspection was undertaken by Vasques-Novoa et al. (2018). They identified a correlation between increased plasma levels of miR-155 and sepsis-related mortality and cardiac injury in humans. Moreover, in mice models, genomic deletion or pharmacological depletion of miR-155 reduced sepsis-associated cardiovascular dysfunction and mortality. Closer examination revealed miR-155 expression in macro- and microvascular endothelial cells and some leukocytes. In vitro examination of the function of miR-155 in microvascular endothelial cells revealed its ability to suppress cytokine and cell adhesion molecule expression. They also found that removal of miR-155 improved AngII reactivity and blunted sepsis-induced systemic and myocardial nitric oxide overproduction. This work showed that the usage of miR-155 is a plausible marker for sepsis outcomes in addition to being a possible therapeutic intervention.

From a different perspective, Wang et al. (2017) published a thorough analysis of circulating endothelial cells (CECs), which can encompass endothelial cells that have detached from the blood vessel (Wang et al. 2017). They examined the role of these CECs and associated miRNAs with sepsis and acute kidney injury (AKI). CECs from septic AKI, non-septic AKI, septic non-AKI patients and healthy volunteers were isolated and their secreted products tested for function. Septic AKI CEC secretions led to cell shrinkage, decreased cell adhesion protein E-cadherin, and cell apoptosis in human kidney tubule cells. Further analysis revealed increased amounts of miR-107 in septic AKI CECs. Inhibition of miR-107 in septic AKI mice through injection recapitulated in vitro findings and preserved the normal renal morphology and decreased the serum creatinine level in mice.

Out of these microRNAs, miR-155 appears to be a robust biomarker compared to other biomarkers such as miR-107 and miR-34a, as it has been found across numerous other publications reviewed here as a sepsis biomarker, although the exact subpopulation of patients may need further refinement (Tacke et al. 2014, 2019). In addition to biomarkers, miRNAs may offer possible therapeutic intervention through the addition of miRNAs which are downregulated in sepsis. As endothelial progenitor cells (EPCs) and CECs can be ex vivo cultured and their exosomes can be harvested as a delivery vehicle, miRNAs such as miR-107 are of interest as a possible therapeutic given that its method of action occurs via circulation.

## MiRNAs and Myeloid-derived suppressor cells in sepsis

Myeloid-derived suppressor cells (MDSC) are a heterogeneous class of immature myeloid cells which act in an immunosuppressive manner and expand during certain inflammatory conditions. Originally described as suppressors of T-cells, it is now appreciated that they interact with numerous immune cells to manifest their suppressive function (Sinha et al. 2007; Sica and Bronte 2007; Schrijver et al. 2019). Although mainly described for their functions in cancer, recent studies have shown that MDSCs play an important role in sepsis (Schrijver et al. 2019). Some groups have reported increases in the number of Gr1^+^CD11b^+^ MDSCs in the bone marrow and spleens of mice during polymicrobial sepsis (Brudecki et al. 2012; Delano et al. 2007). In the acute phase of sepsis, MDSCs are thought to be advantageous in suppressing inflammation (Cuenca and Moldawer 2012). However, if MDSCs continue to expand and infiltrate inflamed tissues, they can induce significant pathophysiology including host immunosuppression that contributes to worsened septic patient outcomes (Hotchkiss et al. 2013).

Within the past decade, the role of miRNAs in the function of MDSC in sepsis has become a small but active area of research. Much of this work has built upon the first published paper in 2014, which focused on two miRNAs in mouse models, miR-21 and miR-181b (McClure et al. 2014). They found an upregulation of miR-21 and miR-181b occurred early in sepsis and was sustained into late sepsis which contributed to the expansion of immature Gr1^+^CD11b^+^ MDSC (McClure et al. 2014). The activity of these miRNAs appears to be upstream of the transcription factor NFI-A, which when deleted, abrogates MDSC immunosuppressive function (Dai et al. 2018). Importantly, in vivo inhibition of the two miRNAs after the initiation of sepsis decreased the bone marrow Gr1^+^CD11b^+^ myeloid progenitors. The possible clinical importance can be seen through the fact that inhibition also led to improved bacterial clearance and reduced late-sepsis mortality by 74%.

Sheng and colleagues examined other mechanisms of action of miR-21 in the regulation of MDSC in sepsis (Sheng et al. 2017). They found miR-375 levels were downregulated but miR-21 was upregulated in human sepsis patients, and their expression were negatively correlated. In mouse models, miR-375 ectopic expression decreased the number of septic Gr1^+^CD11b^+^ MDSC through inhibition of the JAK2-STAT3 pathway (Sheng et al. 2017). The convergence on these findings has been hinted in mouse models of late sepsis, where S100A9 stabilizes the Stat3-C/EBPβ protein complex that activates the miR-21 and miR-181b promoters. In an effort to ensure many of these findings were specific to the MDSC rather than other aspects of in vivo manipulation, Alkhateeb et al. , examined chronic sepsis in mice (Alkhateeb et al. 2019). They performed eloquent experiments reconstituting S100A9 protein to Gr1^+^CD11b^+^ cells from S100A9 knockout mice with late sepsis and found that it restored the Stat3-C/EBPβ protein complex and miR-21 and miR-181b expression, and resulted in the immunosuppressive, MDSC phenotype.

At the clinical level, Hollen and colleagues investigated the function of MDSCs over time in survivors of sepsis who developed chronic critical illness (CCI) and whether such changes were paralleled by complementary miRNA expression patterns (Hollen et al. 2019). Circulating MDSCs from survivors of surgical sepsis were investigated at various intervals over 6 weeks. They found that the number of MDSCs remained for high for 6 weeks after infection but only exhibited a suppressive phenotype at day 14 post septic insult. At the miRNA level, septic patients who developed CCI displayed significant differences in 215 MDSC miRNA levels when compared to patients who rapidly recovered from sepsis across every time point. Overall, this interesting study suggested that the function of MDSC in sepsis may be at least partly regulated via miRNA function.

## Extracellular vesicles of the septic patient

The presence of extracellular vesicles (EVs) offers unique approaches to understanding sepsis. Much of the focus in the literature has been on circulating vesicles in the vasculature, particularly the smaller exosomes which are released to aid in repair of other regions of the vasculature through their contents, often including miRNAs. Moreover, these EVs can be isolated, and their contents analyzed for the purposes of biomarker and therapeutic discovery.

Initial work examining the presence and function of extracellular vesicles in sepsis revealed a possible proinflammatory role. In 2016, Balusu et al. identified a novel way of blood–brain communication activated by peripheral inflammation in mice via secretion of choroid plexus epithelium (CPE)-derived, miRNA-containing EVs (Balusu et al. 2016) The systemic inflammation induced an increase in EVs and associated pro-inflammatory miRNAs, including miR-146a and miR-155, in the CSF. Functionally, EVs enter the brain parenchyma and are taken up by astrocytes and microglia, inducing miRNA target repression and inflammatory gene up-regulation. Further characterization of EVs in mice led to the observation that plasma EVs from septic mice were smaller in size, but higher in number, compared to control mice and injection of mice with septic EVs affected significant peritoneal neutrophil migration (Xu et al. 2018). Septic EVs contained increased amounts of certain miRNAs and proinflammatory markers, and the effects of septic EVs were dampened by miRNA inhibitors against miR-34a, miR-122, and miR-146a. One particularly elegant mouse study from Subramani et al. (2018) tried to understand the functional differences in RBCs in septic mouse models and the role of plasma EVs, rather than examining traditional inflammatory responses (Subramani et al. 2018). They found a significant ex vivo decrease in RBC deformability following sepsis after EVs treatment from septic mice. Moreover, EVs showed distinct molecular profiles in septic mice, particularly a significant increase in expression of miR-6538, and miR-2137.

An in-depth analysis of exosomes in sepsis was a human study profiling total serum, serum exosomes and blood cells to compare miRNAs between the compartments (Reithmair et al. 2017). This group identified numerous miRNAs which were differentially regulated in sepsis, of which miR-27b-3p was present in all three compartments. Further breakdown of the compartments showed miR-199b-5p was a potential early indicator for sepsis and septic shock in the blood cellular compartment, while miR-125b-5p and miR-26b-5p were regulated in exosomes and serum, respectively. The particular role for these miRNAs in their specific compartments and the clinical relevance of modulating these microRNAs has yet to be seen. Another milestone for the clinical usage of exosomes as biomarkers in sepsis was done by Real et al. (2018). They demonstrated that differential exosomal miRNA expression of thirty-five miRNAs were stable up to 7 days in sepsis compared to normal patients. Moreover, this paper also showed that exosomal miRNAs could be clustered according to patient survival and suggested a possible role for dysregulation of the cell cycle in septic patients.

Zhou et al. (2018) took an alternative approach to the therapeutic relevance of exosomes in sepsis (Zhou et al. 2018). EPCs have been well established for the repair of distal damaged tissues through migration but mainly the release of exosomes containing beneficial payloads. They found septic mice treated with EPC exosomes had improved survival, lower lung and renal vascular leakage, reduced liver and kidney dysfunction. The miRNA content of EPC exosomes included abundant miR-126-5p and -3p, known to exist in large quantities in endothelial cells. Importantly, exosomal miR-126-5p and 3p suppressed LPS-induced inflammatory markers in in vitro endothelial cell models. The validity of these findings was supported by inhibition of miR-126-5p and -3p, which abrogated the beneficial effects of EPC exosomes.

## Deregulated miRNAs and their role in COVID-19 disease

The clinical features of severe COVID-19 illness and sepsis have a high degree of overlap. The literature thus far does not present a clear picture as to whether both entities are the same disease in a particular clinical context versus different versions of a similar pro-inflammatory syndrome. As an example, surviving sepsis guidelines have been written specifically for COVID-19 (Alhazzani et al. 2021), yet have a high degree of overlap for managing sepsis in general. Published reports refer to patients with “sepsis caused by severe acute respiratory syndrome coronavirus 2 (SARS-CoV-2)” (Shappell et al. 2022), while others underline the differences between the two diseases (Klaz 2021). At the very least, the two conditions have many parallels at the clinical as well as molecular level suggesting a similar underlying pathogenesis (Olwal et al. 2021; Baghela et al. 2022). In this regard, we have herein sought to understand whether a role for miRNAs has been implicated in the COVID-19 severe illness syndrome.

A pubmed search of “miRNA or small RNA or microRNA” in a predefined general COVID-19 filter (selects only articles related to COVID-19 or SARS-CoV-2) yielded over one thousand results as of July 2022, suggesting an integral role of small RNA biology in COVID-19 pathogenesis. We have not scoured through each article as was done in our original search described above (this was beyond the scope of the current analysis) but noted that many articles studied circulating miRNAs in patients with COVID-19, similar to numerous reports in sepsis (Gonzalo-Calvo et al. 2021; Garcia-Giralt et al. 2022; Li et al. 2021, 2020a; Fayyad-Kazan et al. 2021). One study identified six miRNAs in human plasma associated with COVID-19 illness severity: miR-148-3p, miR-451a, miR-486-5p, miR-192-5p and miR-323a-3p (distinguishing between ward, ICU patients, survivors and non-survivors) (Gonzalo-Calvo et al. 2021). Another report explored differential plasma miRNA expression between patients with COVID-19 and healthy controls, with the following miRNAs showing expression differences: miR-17-5p, miR-142-5p, miR-15a-5p, miR-19a-3p, miR-19b-3p, miR-23a-3p, miR-92a-3p and miR-320a. Circulating miR-369-3p was shown to be associated with acute respiratory distress syndrome in COVID-19 critical illness (Garcia-Giralt et al. 2022).

Since miR-146a and miR-150 surfaced as prominent sepsis-related miRNAs in our review, we specifically searched the literature for their role, if any, in COVID-19 disease. Two reports found circulating miR-150 to be downregulated (as was the case in most sepsis reports—see Table 1) in COVID-19 (Akula et al. 2022; Nicoletti et al. 2022). Sabattinelli et al. looked at miR-146a in the serum of 30 COVID-19 positive patients and 29 healthy controls. Interestingly, they found downregulated miR-146 levels for non-responders to tocilizumab and among the non-responders, lower miR-146a levels in patients with more adverse outcomes (Sabbatinelli et al. 2021). The literature on miRNA levels in COVID-19 supports the notion that small RNA molecules are implicated in its pathogenesis, thus warranting further study on this topic. Furthermore, the similar direction of change of miR-150 and miR-146a in COVID-19 and sepsis may suggest overlapping molecular pathogenesis for both diseases.

## Conclusion

The current review aimed to give a broad understanding of the role of miRNAs systemically involved in the septic disease process. Of note, this review involved a search of articles up to January 2021, thus not including recent publications. Only Pubmed was used as a search engine and in this regard, potentially important information available via other search engines is lacking. Furthermore, organ specific (namely kidney, heart, lung and liver) miRNA involvement in sepsis was not included here but reviewed elsewhere (Manetti et al. 2020; Brandenburger et al. 2018).

After a thorough search of the literature, it is evident that circulating miRNAs in peripheral blood, with their ease of access and mechanistic involvement in the septic process, have great potential for use as diagnostic and prognostic tools. This conclusion arises despite remarkable heterogeneity noted in the miRNA and sepsis literature. However, miRNAs such as miR-146a and miR-150 show consistent changes in human blood across many studies, with strong pathophysiological involvement in the septic process to account for the observations. To improve upon the heterogeneity in reports in future work, a more uniform and accurate methodical strategy is imperative. To this end, the work of Shen et al. has shown improved diagnostic accuracy with miRNAs in serum rather than plasma, use of sepsis-3 criteria versus sepsis-1 or sepsis-2 and non-U6 internal reference for normalization (Shen et al. 2020). Interestingly, a recent report has outlined miRNAs signatures that vary with aging, which may suggest that analyzing miRNA differences should take into account age-matched cohorts for more accurate analyses (Fehlmann et al. 2020). We strongly advocate that, at minimum, future work include an improved normalization strategy beyond U6, and ideally employ software programs like Genorm and Normfinder to identify the best normalizers in the experimental context (Vandesompele et al. 2002; Andersen et al. 2004).

MiRNAs have potential as future treatment strategies for sepsis but therapeutics remain a more complicated issue. In vitro and in vivo work on several miRNAs involved in the septic process, such as miR-146a, have convincingly identified great candidates to be considered for drug delivery, but this is where larger problems arise. The delivery of miRNA therapeutic agents has been complicated by safety concerns and off-target effects (Garzon et al. 2010; Lieberman 2018; Beg et al. 2017). Few miRNA therapeutic agents have progressed to clinical trials with the majority being antisense molecules given that single-stranded oligonucleotides are easier to optimize in vivo, an example being oncogenic miR-155 in lymphoma (Rupaimoole and Slack 2017; Setten et al. 2019; Cheng et al. 2015). Therapeutic replacement of miRNAs has proven challenging because of chemical modifications of the miRNA necessary to avoid nuclease attack, which then interferes with intracellular processing. That being said, a few synthetic miRNA mimics such as miR-29/remlarsen have progressed to initial clinical testing (Rupaimoole and Slack 2017). Indeed, research on delivery systems for small RNA is still ongoing and results in mouse models have become more streamlined and specific, with miRNAs having been shown to be successfully offloaded to specific cell types (Su et al. 2020). We urge the sepsis research community to continue to identify sepsis-specific miRNAs and define their mechanistic action and, in parallel, continue to evolve delivery systems so that, perhaps one day, the two paradigms may neatly intertwine to offer a revolutionary approach in the treatment of this disease.

## Supplementary Information

**Additional file 1: Table S1.** Circulating miRNAs in peripheral blood of patients with sepsis (NB included mainly data validated by RT-qPCR. Microarray/sequencing data shown if validation was not done. Shown in order of publication date. All studies resulted from our Pubmed search as described in the main manuscript). HCs = HCs.

## Data Availability

Not applicable.

## Abbreviations

miRNAs: MicroRNAs
MDSC: Myeloid-derived suppressor cells
EVs: Extracellular vesicles
CCI: Chronic critical illness
CECs: Circulating endothelial cells
AKI: Acute kidney injury
SIRS: Systemic inflammatory response syndrome
HCs: Healthy controls
LPS: Lipopolysaccharide treatment

## References

[CR1] Akula SM, Bolin P, Cook PP. Cellular miR-150-5p may have a crucial role to play in the biology of SARS-CoV-2 infection by regulating nsp10 gene. RNA Biol. 2022;19(1):1–11. 10.1080/15476286.2021.2010959 PMC8786335 34904915

[CR2] Alhazzani W, Evans L, Alshamsi F, Moller MH, Ostermann M, Prescott HC (2021). surviving sepsis campaign guidelines on the management of adults with coronavirus disease 2019 (COVID-19) in the ICU: first update. Crit Care Med.

[CR3] Alkhateeb T, Kumbhare A, Bah I, Youssef D, Yao ZQ, McCall CE (2019). S100A9 maintains myeloid-derived suppressor cells in chronic sepsis by inducing miR-21 and miR-181b. Mol Immunol.

[CR4] Andersen CL, Jensen JL, Orntoft TF (2004). Normalization of real-time quantitative reverse transcription-PCR data: a model-based variance estimation approach to identify genes suited for normalization, applied to bladder and colon cancer data sets. Cancer Res.

[CR5] Angus DC, van der Poll T (2013). Severe sepsis and septic shock. N Engl J Med.

[CR6] Angus DC, Linde-Zwirble WT, Lidicker J, Clermont G, Carcillo J, Pinsky MR (2001). Epidemiology of severe sepsis in the United States: analysis of incidence, outcome, and associated costs of care. Crit Care Med.

[CR7] Baghela A, Pena OM, Lee AH, Baquir B, Falsafi R, An A (2022). Predicting sepsis severity at first clinical presentation: the role of endotypes and mechanistic signatures. EBioMedicine.

[CR8] Balusu S, Van Wonterghem E, De Rycke R, Raemdonck K, Stremersch S, Gevaert K (2016). Identification of a novel mechanism of blood-brain communication during peripheral inflammation via choroid plexus-derived extracellular vesicles. EMBO Mol Med.

[CR9] Bartel DP (2004). MicroRNAs: genomics, biogenesis, mechanism, and function. Cell.

[CR10] Beg MS, Brenner AJ, Sachdev J, Borad M, Kang YK, Stoudemire J (2017). Phase I study of MRX34, a liposomal miR-34a mimic, administered twice weekly in patients with advanced solid tumors. Invest New Drugs.

[CR11] Benz F, Roderburg C, Vargas Cardenas D, Vucur M, Gautheron J, Koch A (2013). U6 is unsuitable for normalization of serum miRNA levels in patients with sepsis or liver fibrosis. Exp Mol Med.

[CR12] Benz F, Tacke F, Luedde M, Trautwein C, Luedde T, Koch A (2015). Circulating microRNA-223 serum levels do not predict sepsis or survival in patients with critical illness. Dis Markers.

[CR13] Boldin MP, Taganov KD, Rao DS, Yang L, Zhao JL, Kalwani M (2011). miR-146a is a significant brake on autoimmunity, myeloproliferation, and cancer in mice. J Exp Med.

[CR14] Boyd JH, Russell JA, Fjell CD (2014). The meta-genome of sepsis: host genetics, pathogens and the acute immune response. J Innate Immun.

[CR15] Brandenburger T, Salgado Somoza A, Devaux Y, Lorenzen JM (2018). Noncoding RNAs in acute kidney injury. Kidney Int.

[CR16] Braza-Boils A, Barwari T, Gutmann C, Thomas MR, Judge HM, Joshi A, et al. (Circulating microRNA levels indicate platelet and leukocyte activation in endotoxemia despite platelet P2Y12 inhibition. Int J Mol Sci. 2020;21(8). 10.3390/ijms21082897 PMC7215420 32326325

[CR17] Brudecki L, Ferguson DA, McCall CE, El Gazzar M (2012). Myeloid-derived suppressor cells evolve during sepsis and can enhance or attenuate the systemic inflammatory response. Infect Immun.

[CR18] Brudecki L, Ferguson DA, McCall CE, El Gazzar M (2013). MicroRNA-146a and RBM4 form a negative feed-forward loop that disrupts cytokine mRNA translation following TLR4 responses in human THP-1 monocytes. Immunol Cell Biol.

[CR19] Cao X, Zhang C, Zhang X, Chen Y, Zhang H (2019). MiR-145 negatively regulates TGFBR2 signaling responsible for sepsis-induced acute lung injury. Biomed Pharmacother.

[CR20] Caserta S, Kern F, Cohen J, Drage S, Newbury SF, Llewelyn MJ (2016). Circulating plasma microRNAs can differentiate human sepsis and systemic inflammatory response syndrome (SIRS). Sci Rep.

[CR21] Cavaillon JM, Adib-Conquy M (2006). Bench-to-bedside review: endotoxin tolerance as a model of leukocyte reprogramming in sepsis. Crit Care.

[CR22] Cecconi M, Evans L, Levy M, Rhodes A (2018). Sepsis and septic shock. Lancet.

[CR23] Chen X, Song D. LPS promotes the progression of sepsis by activation of lncRNA HULC/miR-204–5p/TRPM7 network in HUVECs. Biosci Rep. 2020;40(6). 10.1042/BSR20200740 PMC7295636 32484206

[CR24] Chen X, Chen Y, Dai L, Wang N. MiR-96–5p alleviates inflammatory responses by targeting NAMPT and regulating the NF-kappaB pathway in neonatal sepsis. Biosci Rep. 2020a;40(7). 10.1042/BSR20201267 PMC7335832 32618342

[CR25] Chen W, Liu L, Yang J, Wang Y (2020). MicroRNA-146b correlates with decreased acute respiratory distress syndrome risk, reduced disease severity, and lower 28-day mortality in sepsis patients. J Clin Lab Anal.

[CR26] Chen L, Xie W, Wang L, Zhang X, Liu E, Kou Q (2020). MiRNA-133a aggravates inflammatory responses in sepsis by targeting SIRT1. Int Immunopharmacol.

[CR27] Chen L, Yu L, Zhang R, Zhu L, Shen W (2020). Correlation of microRNA-146a/b with disease risk, biochemical indices, inflammatory cytokines, overall disease severity, and prognosis of sepsis. Medicine (baltimore).

[CR28] Cheng CJ, Bahal R, Babar IA, Pincus Z, Barrera F, Liu C (2015). MicroRNA silencing for cancer therapy targeted to the tumour microenvironment. Nature.

[CR29] Chu M, Qin S, Wu R, Zhou X, Tang X, Zhang S (2016). Role of MiR-126a-3p in endothelial injury in endotoxic mice. Crit Care Med.

[CR30] Colbert JF, Schmidt EP (2016). Endothelial and microcirculatory function and dysfunction in sepsis. Clin Chest Med.

[CR31] Cuenca AG, Moldawer LL (2012). Myeloid-derived suppressor cells in sepsis: friend or foe?. Intensive Care Med.

[CR32] Dai J, Kumbhare A, Williams DA, Youssef D, Yao ZQ, McCall CE (2018). Nfia deletion in myeloid cells blocks expansion of myeloid-derived suppressor cells during sepsis. Innate Immun.

[CR33] de Gonzalo-Calvo D, Benitez ID, Pinilla L, Carratala A, Moncusi-Moix A, Gort-Paniello C (2021). Circulating microRNA profiles predict the severity of COVID-19 in hospitalized patients. Transl Res.

[CR34] Delano MJ, Scumpia PO, Weinstein JS, Coco D, Nagaraj S, Kelly-Scumpia KM (2007). MyD88-dependent expansion of an immature GR-1(+)CD11b(+) population induces T cell suppression and Th2 polarization in sepsis. J Exp Med.

[CR35] Doxaki C, Kampranis SC, Eliopoulos AG, Spilianakis C, Tsatsanis C (2015). Coordinated regulation of miR-155 and miR-146a genes during induction of endotoxin tolerance in macrophages. J Immunol.

[CR36] Evans L, Rhodes A, Alhazzani W, Antonelli M, Coopersmith CM, French C (2021). Surviving sepsis campaign: international guidelines for management of sepsis and septic shock 2021. Crit Care Med.

[CR37] Fatmi A, Rebiahi SA, Chabni N, Zerrouki H, Azzaoui H, Elhabiri Y (2020). miRNA-23b as a biomarker of culture-positive neonatal sepsis. Mol Med.

[CR38] Fayyad-Kazan M, Makki R, Skafi N, El Homsi M, Hamade A, El Majzoub R (2021). Circulating miRNAs: potential diagnostic role for coronavirus disease 2019 (COVID-19). Infect Genet Evol.

[CR39] Fehlmann T, Lehallier B, Schaum N, Hahn O, Kahraman M, Li Y (2020). Common diseases alter the physiological age-related blood microRNA profile. Nat Commun.

[CR40] Formosa A, Markert EK, Lena AM, Italiano D, Finazzi-Agro E, Levine AJ (2014). MicroRNAs, miR-154, miR-299-5p, miR-376a, miR-376c, miR-377, miR-381, miR-487b, miR-485-3p, miR-495 and miR-654-3p, mapped to the 14q32.31 locus, regulate proliferation, apoptosis, migration and invasion in metastatic prostate cancer cells. Oncogene.

[CR41] Gao N, Dong L (2017). MicroRNA-146 regulates the inflammatory cytokines expression in vascular endothelial cells during sepsis. Pharmazie.

[CR42] Gao M, Yu T, Liu D, Shi Y, Yang P, Zhang J (2021). Sepsis plasma-derived exosomal miR-1-3p induces endothelial cell dysfunction by targeting SERP1. Clin Sci (lond).

[CR43] Garcia-Giralt N, Du J, Marin-Corral J, Bodalo-Torruella M, Blasco-Hernando F, Munoz-Bermudez R (2022). Circulating microRNA profiling is altered in the acute respiratory distress syndrome related to SARS-CoV-2 infection. Sci Rep.

[CR44] Garzon R, Marcucci G, Croce CM (2010). Targeting microRNAs in cancer: rationale, strategies and challenges. Nat Rev Drug Discov.

[CR45] Ge Y, Huang M, Ma YF (2017). The effects of microRNA-34a regulating Notch-1/NF-kappaB signaling pathway on lipopolysaccharide-induced human umbilical vein endothelial cells. World J Emerg Med.

[CR46] Ge QM, Huang CM, Zhu XY, Bian F, Pan SM (2017). Differentially expressed miRNAs in sepsis-induced acute kidney injury target oxidative stress and mitochondrial dysfunction pathways. PLoS ONE.

[CR47] Giza DE, Fuentes-Mattei E, Bullock MD, Tudor S, Goblirsch MJ, Fabbri M (2016). Cellular and viral microRNAs in sepsis: mechanisms of action and clinical applications. Cell Death Differ.

[CR48] Goodwin AJ, Guo C, Cook JA, Wolf B, Halushka PV, Fan H (2015). Plasma levels of microRNA are altered with the development of shock in human sepsis: an observational study. Crit Care.

[CR49] Guo C, Goodwin A, Buie JNJ, Cook J, Halushka P, Argraves K (2016). A stromal cell-derived factor 1alpha analogue improves endothelial cell function in lipopolysaccharide-induced acute respiratory distress syndrome. Mol Med.

[CR50] Han Y, Dai QC, Shen HL, Zhang XW (2016). Diagnostic value of elevated serum miRNA-143 levels in sepsis. J Int Med Res.

[CR51] Hellemans J, Vandesompele J (2014). Selection of reliable reference genes for RT-qPCR analysis. Methods Mol Biol.

[CR52] Heng J, Wu D, Lu S, Zhao Y (2020). miR-106a targets anoctamin 1 (ANO1) to regulate lipopolysaccharide (LPS)-induced inflammatory response in macrophages. Med Sci Monit.

[CR53] Hermann S, Brandes F, Kirchner B, Buschmann D, Borrmann M, Klein M (2020). Diagnostic potential of circulating cell-free microRNAs for community-acquired pneumonia and pneumonia-related sepsis. J Cell Mol Med.

[CR54] Hirschberger S, Hubner M, Strauss G, Effinger D, Bauer M, Weis S (2019). Identification of suitable controls for miRNA quantification in T-cells and whole blood cells in sepsis. Sci Rep.

[CR55] Hollen MK, Stortz JA, Darden D, Dirain ML, Nacionales DC, Hawkins RB (2019). Myeloid-derived suppressor cell function and epigenetic expression evolves over time after surgical sepsis. Crit Care.

[CR56] Hotchkiss RS, Monneret G, Payen D (2013). Sepsis-induced immunosuppression: from cellular dysfunctions to immunotherapy. Nat Rev Immunol.

[CR57] How CK, Hou SK, Shih HC, Huang MS, Chiou SH, Lee CH (2015). Expression profile of MicroRNAs in gram-negative bacterial sepsis. Shock.

[CR58] Huang J, Sun Z, Yan W, Zhu Y, Lin Y, Chen J (2014). Identification of microRNA as sepsis biomarker based on miRNAs regulatory network analysis. Biomed Res Int.

[CR59] Ince C, Mayeux PR, Nguyen T, Gomez H, Kellum JA, Ospina-Tascon GA (2016). The endothelium in sepsis. Shock.

[CR60] Ivey KN, Srivastava D (2015). microRNAs as developmental regulators. Cold Spring Harb Perspect Biol.

[CR61] Joffre J, Hellman J, Ince C, Ait-Oufella H (2020). Endothelial responses in sepsis. Am J Respir Crit Care Med.

[CR62] Jones Buie JN, Zhou Y, Goodwin AJ, Cook JA, Vournakis J, Demcheva M (2019). Application of deacetylated Poly-N-acetyl glucosamine nanoparticles for the delivery of miR-126 for the treatment of cecal ligation and puncture-induced sepsis. Inflammation.

[CR63] Karam RA, Zidan HE, Karam NA, Abdel Rahman DM, El-Seifi OS (2019). Diagnostic and prognostic significance of serum miRNA-146-a expression in Egyptian children with sepsis in a pediatric intensive care unit. J Gene Med.

[CR64] Kirschner MB, Kao SC, Edelman JJ, Armstrong NJ, Vallely MP, van Zandwijk N (2011). Haemolysis during sample preparation alters microRNA content of plasma. PLoS ONE.

[CR65] Klaz I. Treating sepsis in COVID-19 patients online: Wolters Kluwer; 2021. https://www.wolterskluwer.com/en/expert-insights/treating-sepsis-in-covid-19-patients.

[CR66] Kumar A, Roberts D, Wood KE, Light B, Parrillo JE, Sharma S (2006). Duration of hypotension before initiation of effective antimicrobial therapy is the critical determinant of survival in human septic shock. Crit Care Med.

[CR67] Labbaye C, Testa U (2012). The emerging role of MIR-146A in the control of hematopoiesis, immune function and cancer. J Hematol Oncol.

[CR68] Lee RC, Feinbaum RL, Ambros V (1993). The *C. elegans* heterochronic gene lin-4 encodes small RNAs with antisense complementarity to lin-14. Cell.

[CR69] Li C, Hu X, Li L, Li JH (2020). Differential microRNA expression in the peripheral blood from human patients with COVID-19. J Clin Lab Anal.

[CR70] Li Z, Yi N, Chen R, Meng Y, Wang Y, Liu H (2020). miR-29b-3p protects cardiomyocytes against endotoxin-induced apoptosis and inflammatory response through targeting FOXO3A. Cell Signal.

[CR71] Li S, Zhao D, Cui J, Wang L, Ma X, Li Y (2020). Correlation of microRNA-125a/b with acute respiratory distress syndrome risk and prognosis in sepsis patients. J Clin Lab Anal.

[CR72] Li CX, Chen J, Lv SK, Li JH, Li LL, Hu X (2021). Whole-transcriptome RNA sequencing reveals significant differentially expressed mRNAs, miRNAs, and lncRNAs and related regulating biological pathways in the peripheral blood of COVID-19 patients. Mediators Inflamm.

[CR73] Liang G, Wu Y, Guan Y, Dong Y, Jiang L, Mao G (2020). The correlations between the serum expression of miR-206 and the severity and prognosis of sepsis. Ann Palliat Med.

[CR74] Lieberman J (2018). Tapping the RNA world for therapeutics. Nat Struct Mol Biol.

[CR75] Lin R, Hu H, Li L, Chen G, Luo L, Rao P (2020). The potential of microRNA-126 in predicting disease risk, mortality of sepsis, and its correlation with inflammation and sepsis severity. J Clin Lab Anal.

[CR76] Liu J, Shi K, Chen M, Xu L, Hong J, Hu B (2015). Elevated miR-155 expression induces immunosuppression via CD39(+) regulatory T-cells in sepsis patient. Int J Infect Dis.

[CR77] Liu W, Geng F, Yu L (2020). Long non-coding RNA MALAT1/microRNA 125a axis presents excellent value in discriminating sepsis patients and exhibits positive association with general disease severity, organ injury, inflammation level, and mortality in sepsis patients. J Clin Lab Anal.

[CR78] Liu G, Liu W, Guo J (2020). Clinical significance of miR-181a in patients with neonatal sepsis and its regulatory role in the lipopolysaccharide-induced inflammatory response. Exp Ther Med.

[CR79] Liu D, Wang Z, Wang H, Ren F, Li Y, Zou S (2020). The protective role of miR-223 in sepsis-induced mortality. Sci Rep.

[CR80] Liu Z, Yang D, Gao J, Xiang X, Hu X, Li S (2020). Discovery and validation of miR-452 as an effective biomarker for acute kidney injury in sepsis. Theranostics.

[CR81] Liu L, Liu F, Sun Z, Peng Z, You T, Yu Z (2020). LncRNA NEAT1 promotes apoptosis and inflammation in LPS-induced sepsis models by targeting miR-590-3p. Exp Ther Med.

[CR82] Ma Y, Vilanova D, Atalar K, Delfour O, Edgeworth J, Ostermann M (2013). Genome-wide sequencing of cellular microRNAs identifies a combinatorial expression signature diagnostic of sepsis. PLoS ONE.

[CR83] Ma Y, Liu Y, Hou H, Yao Y, Meng H (2018). MiR-150 predicts survival in patients with sepsis and inhibits LPS-induced inflammatory factors and apoptosis by targeting NF-kappaB1 in human umbilical vein endothelial cells. Biochem Biophys Res Commun.

[CR84] Manetti AC, Maiese A, Paolo MD, De Matteis A, La Russa R, Turillazzi E, et al. (MicroRNAs and sepsis-induced cardiac dysfunction: a systematic review. Int J Mol Sci. 2020;22(1). 10.3390/ijms22010321 PMC7794809 33396834

[CR85] McClure C, Brudecki L, Ferguson DA, Yao ZQ, Moorman JP, McCall CE (2014). MicroRNA 21 (miR-21) and miR-181b couple with NFI-A to generate myeloid-derived suppressor cells and promote immunosuppression in late sepsis. Infect Immun.

[CR86] Mohnle P, Schutz SV, van der Heide V, Hubner M, Luchting B, Sedlbauer J (2015). MicroRNA-146a controls Th1-cell differentiation of human CD4+ T lymphocytes by targeting PRKCepsilon. Eur J Immunol.

[CR87] Mohnle P, Hirschberger S, Hinske LC, Briegel J, Hubner M, Weis S (2018). MicroRNAs 143 and 150 in whole blood enable detection of T-cell immunoparalysis in sepsis. Mol Med.

[CR88] Na L, Ding H, Xing E, Zhang Y, Gao J, Liu B (2020). The predictive value of microRNA-21 for sepsis risk and its correlation with disease severity, systemic inflammation, and 28-day mortality in sepsis patients. J Clin Lab Anal.

[CR89] Nahid MA, Pauley KM, Satoh M, Chan EK (2009). miR-146a is critical for endotoxin-induced tolerance: implication in innate immunity. J Biol Chem.

[CR90] Nicoletti AS, Visacri MB, da Ronda C, Vasconcelos P, Quintanilha JCF, de Souza RN (2022). Differentially expressed plasmatic microRNAs in Brazilian patients with Coronavirus disease 2019 (COVID-19): preliminary results. Mol Biol Rep.

[CR91] Olwal CO, Nganyewo NN, Tapela K, Djomkam Zune AL, Owoicho O, Bediako Y (2021). Parallels in sepsis and COVID-19 conditions: implications for managing severe COVID-19. Front Immunol.

[CR92] Pfeiffer D, Rossmanith E, Lang I, Falkenhagen D (2017). miR-146a, miR-146b, and miR-155 increase expression of IL-6 and IL-8 and support HSP10 in an In vitro sepsis model. PLoS ONE.

[CR93] Pritchard CC, Kroh E, Wood B, Arroyo JD, Dougherty KJ, Miyaji MM (2012). Blood cell origin of circulating microRNAs: a cautionary note for cancer biomarker studies. Cancer Prev Res (phila).

[CR94] Qin Y, Guo X, Yu Y, Dong S, Yan Y, Bian X (2020). Screening key genes and microRNAs in sepsis by RNA-sequencing. J Chin Med Assoc.

[CR95] Rahmel T, Schafer ST, Frey UH, Adamzik M, Peters J (2018). Increased circulating microRNA-122 is a biomarker for discrimination and risk stratification in patients defined by sepsis-3 criteria. PLoS ONE.

[CR96] Rajput C, Tauseef M, Farazuddin M, Yazbeck P, Amin MR, Avin Br V (2016). MicroRNA-150 suppression of angiopoetin-2 generation and signaling is crucial for resolving vascular injury. Arterioscler Thromb Vasc Biol.

[CR97] Ranieri VM, Thompson BT, Barie PS, Dhainaut JF, Douglas IS, Finfer S (2012). Drotrecogin alfa (activated) in adults with septic shock. N Engl J Med.

[CR98] Real JM, Ferreira LRP, Esteves GH, Koyama FC, Dias MVS, Bezerra-Neto JE (2018). Exosomes from patients with septic shock convey miRNAs related to inflammation and cell cycle regulation: new signaling pathways in sepsis?. Crit Care.

[CR99] Reithmair M, Buschmann D, Marte M, Kirchner B, Hagl D, Kaufmann I (2017). Cellular and extracellular miRNAs are blood-compartment-specific diagnostic targets in sepsis. J Cell Mol Med.

[CR100] Roderburg C, Luedde M, Vargas Cardenas D, Vucur M, Scholten D, Frey N (2013). Circulating microRNA-150 serum levels predict survival in patients with critical illness and sepsis. PLoS ONE.

[CR101] Roderburg C, Benz F, Koch A, Loosen SH, Spehlmann M, Luedde M, et al. (A combined score of circulating miRNAs allows outcome prediction in critically ill patients. J Clin Med. 2019;8(10). 10.3390/jcm8101644 PMC6832199 31601014

[CR102] Rupaimoole R, Slack FJ (2017). MicroRNA therapeutics: towards a new era for the management of cancer and other diseases. Nat Rev Drug Discov.

[CR103] Sabbatinelli J, Giuliani A, Matacchione G, Latini S, Laprovitera N, Pomponio G (2021). Decreased serum levels of the inflammaging marker miR-146a are associated with clinical non-response to tocilizumab in COVID-19 patients. Mech Ageing Dev.

[CR104] Schlosser K, McIntyre LA, White RJ, Stewart DJ (2015). Customized internal reference controls for improved assessment of circulating microRNAs in disease. PLoS ONE.

[CR105] Schmidt WM, Spiel AO, Jilma B, Wolzt M, Muller M (2009). In vivo profile of the human leukocyte microRNA response to endotoxemia. Biochem Biophys Res Commun.

[CR106] Schrijver IT, Theroude C, Roger T (2019). Myeloid-derived suppressor cells in sepsis. Front Immunol.

[CR107] Setten RL, Rossi JJ, Han SP (2019). The current state and future directions of RNAi-based therapeutics. Nat Rev Drug Discov.

[CR108] Shao Y, Li J, Cai Y, Xie Y, Ma G, Li Y (2014). The functional polymorphisms of miR-146a are associated with susceptibility to severe sepsis in the Chinese population. Mediators Inflamm.

[CR109] Shappell CN, Klompas M, Kanjilal S, Chan C, Rhee C (2022). Prevalence, clinical characteristics, and outcomes of sepsis caused by severe acute respiratory syndrome coronavirus 2 versus other pathogens in hospitalized patients with COVID-19. Crit Care Explor.

[CR110] Shen X, Zhang J, Huang Y, Tong J, Zhang L, Zhang Z (2020). Accuracy of circulating microRNAs in diagnosis of sepsis: a systematic review and meta-analysis. J Intensive Care.

[CR111] Sheng B, Zhao L, Zang X, Zhen J, Chen W (2017). miR-375 ameliorates sepsis by downregulating miR-21 level via inhibiting JAK2-STAT3 signaling. Biomed Pharmacother.

[CR112] Sica A, Bronte V (2007). Altered macrophage differentiation and immune dysfunction in tumor development. J Clin Invest.

[CR113] Singer M, Deutschman CS, Seymour CW, Shankar-Hari M, Annane D, Bauer M (2016). The Third International Consensus Definitions for Sepsis and Septic Shock (Sepsis-3). JAMA.

[CR114] Sinha P, Clements VK, Bunt SK, Albelda SM, Ostrand-Rosenberg S (2007). Cross-talk between myeloid-derived suppressor cells and macrophages subverts tumor immunity toward a type 2 response. J Immunol.

[CR115] Su YL, Wang X, Mann M, Adamus TP, Wang D, Moreira DF (2020). Myeloid cell-targeted miR-146a mimic inhibits NF-kappaB-driven inflammation and leukemia progression in vivo. Blood.

[CR116] Subramani K, Raju SP, Chu X, Warren M, Pandya CD, Hoda N (2018). Effect of plasma-derived extracellular vesicles on erythrocyte deformability in polymicrobial sepsis. Int Immunopharmacol.

[CR117] Tacke F, Roderburg C, Benz F, Cardenas DV, Luedde M, Hippe HJ (2014). Levels of circulating miR-133a are elevated in sepsis and predict mortality in critically ill patients. Crit Care Med.

[CR118] Tacke F, Spehlmann ME, Vucur M, Benz F, Luedde M, Cardenas DV (2019). miR-155 predicts long-term mortality in critically ill patients younger than 65 years. Mediators Inflamm.

[CR119] Tano N, Kim HW, Ashraf M (2011). microRNA-150 regulates mobilization and migration of bone marrow-derived mononuclear cells by targeting Cxcr4. PLoS ONE.

[CR120] Testa U, Pelosi E, Castelli G, Labbaye C. miR-146 and miR-155: Two Key Modulators of Immune Response and Tumor Development. Noncoding RNA. 2017;3(3). 10.3390/ncrna3030022 PMC5831915 29657293

[CR121] Tili E, Michaille JJ, Cimino A, Costinean S, Dumitru CD, Adair B (2007). Modulation of miR-155 and miR-125b levels following lipopolysaccharide/TNF-alpha stimulation and their possible roles in regulating the response to endotoxin shock. J Immunol.

[CR122] Tudor S, Giza DE, Lin HY, Fabris L, Yoshiaki K, D'Abundo L (2014). Cellular and Kaposi's sarcoma-associated herpes virus microRNAs in sepsis and surgical trauma. Cell Death Dis.

[CR123] van Zandwijk N, Pavlakis N, Kao SC, Linton A, Boyer MJ, Clarke S (2017). Safety and activity of microRNA-loaded minicells in patients with recurrent malignant pleural mesothelioma: a first-in-man, phase 1, open-label, dose-escalation study. Lancet Oncol.

[CR124] Vandesompele J, De Preter K, Pattyn F, Poppe B, Van Roy N, De Paepe A (2002). Accurate normalization of real-time quantitative RT-PCR data by geometric averaging of multiple internal control genes. Genome Biol.

[CR125] Vasilescu C, Rossi S, Shimizu M, Tudor S, Veronese A, Ferracin M (2009). MicroRNA fingerprints identify miR-150 as a plasma prognostic marker in patients with sepsis. PLoS ONE.

[CR126] Vasques-Novoa F, Laundos TL, Cerqueira RJ, Quina-Rodrigues C, Soares-Dos-Reis R, Baganha F (2018). MicroRNA-155 amplifies nitric oxide/cGMP signaling and impairs vascular angiotensin II reactivity in septic shock. Crit Care Med.

[CR127] Wang JF, Yu ML, Yu G, Bian JJ, Deng XM, Wan XJ (2010). Serum miR-146a and miR-223 as potential new biomarkers for sepsis. Biochem Biophys Res Commun.

[CR128] Wang HJ, Zhang PJ, Chen WJ, Feng D, Jia YH, Xie LX (2012). Four serum microRNAs identified as diagnostic biomarkers of sepsis. J Trauma Acute Care Surg.

[CR129] Wang H, Zhang P, Chen W, Feng D, Jia Y, Xie L (2012). Serum microRNA signatures identified by Solexa sequencing predict sepsis patients' mortality: a prospective observational study. PLoS ONE.

[CR130] Wang H, Meng K, Chen W, Feng D, Jia Y, Xie L (2012). Serum miR-574-5p: a prognostic predictor of sepsis patients. Shock.

[CR131] Wang H, Zhang P, Chen W, Feng D, Jia Y, Xie LX (2012). Evidence for serum miR-15a and miR-16 levels as biomarkers that distinguish sepsis from systemic inflammatory response syndrome in human subjects. Clin Chem Lab Med.

[CR132] Wang L, Wang HC, Chen C, Zeng J, Wang Q, Zheng L (2013). Differential expression of plasma miR-146a in sepsis patients compared with non-sepsis-SIRS patients. Exp Ther Med.

[CR133] Wang HJ, Zhang PJ, Chen WJ, Jie D, Dan F, Jia YH (2013). Characterization and Identification of novel serum microRNAs in sepsis patients with different outcomes. Shock.

[CR134] Wang H, Yu B, Deng J, Jin Y, Xie L (2014). Serum miR-122 correlates with short-term mortality in sepsis patients. Crit Care.

[CR135] Wang S, Zhang Z, Wang J, Miao H (2017). MiR-107 induces TNF-alpha secretion in endothelial cells causing tubular cell injury in patients with septic acute kidney injury. Biochem Biophys Res Commun.

[CR136] Wang Q, Feng Q, Zhang Y, Zhou S, Chen H (2020). Decreased microRNA 103 and microRNA 107 predict increased risks of acute respiratory distress syndrome and 28-day mortality in sepsis patients. Medicine (baltimore).

[CR137] Wang Y, Xu Z, Yue D, Zeng Z, Yuan W, Xu K (2020). Linkage of lncRNA CRNDE sponging miR-181a-5p with aggravated inflammation underlying sepsis. Innate Immun.

[CR138] Wei B, Yu L (2020). Circular RNA PRKCI and microRNA-545 relate to sepsis risk, disease severity and 28-day mortality. Scand J Clin Lab Invest.

[CR139] Wu X, Yang J, Yu L, Long D (2018). Plasma miRNA-223 correlates with risk, inflammatory markers as well as prognosis in sepsis patients. Medicine (baltimore).

[CR140] Xu J, Feng Y, Jeyaram A, Jay SM, Zou L, Chao W (2018). Circulating plasma extracellular vesicles from septic mice induce inflammation via microRNA- and TLR7-dependent mechanisms. J Immunol.

[CR141] Xu H, Liu X, Ni H (2020). Clinical significance of miR-19b-3p in patients with sepsis and its regulatory role in the LPS-induced inflammatory response. Eur J Med Res.

[CR142] Xue Z, Xi Q, Liu H, Guo X, Zhang J, Zhang Z (2019). miR-21 promotes NLRP3 inflammasome activation to mediate pyroptosis and endotoxic shock. Cell Death Dis.

[CR143] Yang M, Zhao L, Sun M (2020). Diagnostic value of miR-103 in patients with sepsis and noninfectious SIRS and its regulatory role in LPS-induced inflammatory response by targeting TLR4. Int J Genomics.

[CR144] Yang Y, Yang L, Liu Z, Wang Y, Yang J (2020). Long noncoding RNA NEAT 1 and its target microRNA-125a in sepsis: Correlation with acute respiratory distress syndrome risk, biochemical indexes, disease severity, and 28-day mortality. J Clin Lab Anal.

[CR145] Yang W, Luo X, Liu Y, Xiong J, Xia H, Liu Y (2020). Potential role of lncRNA HULC/miR1283p/RAC1 axis in the inflammatory response during LPSinduced sepsis in HMEC1 cells. Mol Med Rep.

[CR146] Yousefpouran S, Mostafaei S, Manesh PV, Iranifar E, Bokharaei-Salim F, Nahand JS (2020). The assessment of selected MiRNAs profile in HIV, HBV, HCV, HIV/HCV, HIV/HBV Co-infection and elite controllers for determination of biomarker. Microb Pathog.

[CR147] Zhang Y, Kim MS, Jia B, Yan J, Zuniga-Hertz JP, Han C (2017). Hypothalamic stem cells control ageing speed partly through exosomal miRNAs. Nature.

[CR148] Zhang W, Jia J, Liu Z, Si D, Ma L, Zhang G (2019). Circulating microRNAs as biomarkers for Sepsis secondary to pneumonia diagnosed via Sepsis 3.0. BMC Pulm Med..

[CR149] Zhang Y, Li M, Bao L, Hu P (2019). A case-control study on the relationship between miRNAs single nucleotide polymorphisms and sepsis risk. Medicine (baltimore).

[CR150] Zhao JL, Rao DS, Boldin MP, Taganov KD, O'Connell RM, Baltimore D (2011). NF-kappaB dysregulation in microRNA-146a-deficient mice drives the development of myeloid malignancies. Proc Natl Acad Sci U S A.

[CR151] Zhen J, Chen W (2018). MiR-142 inhibits cecal ligation and puncture (CLP)-induced inflammation via inhibiting PD-L1 expression in macrophages and improves survival in septic mice. Biomed Pharmacother.

[CR152] Zhou YP, Xia Q (2020). Inhibition of miR-103a-3p suppresses lipopolysaccharide-induced sepsis and liver injury by regulating FBXW7 expression. Cell Biol Int.

[CR153] Zhou J, Chaudhry H, Zhong Y, Ali MM, Perkins LA, Owens WB (2015). Dysregulation in microRNA expression in peripheral blood mononuclear cells of sepsis patients is associated with immunopathology. Cytokine.

[CR154] Zhou Y, Li P, Goodwin AJ, Cook JA, Halushka PV, Chang E (2018). Exosomes from endothelial progenitor cells improve the outcome of a murine model of sepsis. Mol Ther.

[CR155] Zhu X (2020). MiR-125b but not miR-125a is upregulated and exhibits a trend to correlate with enhanced disease severity, inflammation, and increased mortality in sepsis patients. J Clin Lab Anal.

