# Supplementary material for: Role of miRNA dysregulation in sepsis
Source: Mol Med. 2022 Aug 19;28:99. doi: 10.1186/s10020-022-00527-z (PMC9389495; doi:10.1186/s10020-022-00527-z)
Supplement: Supplementary file 1 — Additional file 1: Table S1. Circulating miRNAs in peripheral blood of patients with sepsis (NB included mainly data validated by RT-qPCR. Microarray/sequencing data shown if validation was not done. Shown in order of publication date. All studies resulted from our Pubmed search as described in the main manuscript). HCs = HCs. [file 10020_2022_527_MOESM1_ESM.docx]

| **Supplementary table 1:** Circulating miRNAs in peripheral blood of patients with sepsis (NB included mainly data validated by RT-qPCR. Microarray/sequencing data shown if validation was not done. Shown in order of publication date. All studies resulted from our Pubmed search as described in the main manuscript). HCs = HCs | | | | | | |
| --- | --- | --- | --- | --- | --- | --- |
| **miRNAs studied** | **Sample type** | **Population and sample size** | **Direction of fold change in sepsis** | **Technique** | **Normalization (if RT-qPCR)** | **Reference** |
| miR-1-3p | Plasma exosomes | 3 patients with sepsis  3 HCs | Increased | RT-qPCR | U6 | (1) |
| Many – see manuscript | Whole blood leukocytes | 156 patients with sepsis  82 HCs | 635 significantly altered small non-coding RNAs | Next generation microarray analysis | -- | (2) |
| miR-452 | Serum | 97 patients with sepsis  10 HCs | Upregulated | RT-qPCR | Cel-39 | (3) |
| Many – see manuscript | Peripheral blood | 12 patients with sepsis  3 HCs | (see manuscript for details)  11 upregulated miRNAs  851 downregulated | In silico analysis of data series in GEO database | -- | (4) |
| miR-128-3p | Serum | 110 patients with sepsis  100 HCs | Downregulated | RT-qPCR | U6 | (5) |
| miR-223 | White blood cells from peripheral blood | 143 patients with sepsis  44 HCs | Upregulated in sepsis  Upregulated in survivors compared to non-survivors | RT-qPCR | 5S rRNA | (6) |
| miR106a | Serum | 31 patients with sepsis  31 HCs | Upregulated | RT-qPCR | U6 | (7) |
| miR-23b | “cell free supernatant” | 48 newborns with clinical features of sepsis | Upregulated in early onset sepsis compared to hemoculture negative  Downregulated in late onset sepsis, further downregulated in patients who died | RT-qPCR | U6 | (8) |
| miR-545 | Plasma | 121 patients with sepsis  60 HCs | Upregulated | RT-qPCR | U6 | (9) |
| miR-223-3p  miR-26b-5p  miR-193-3p | Serum | 12 septic intensive care patients  12 non-septic intensive care patients  12 HCs | miR-223-3p: downregulated in septic ICU compared to HC but no change from septic to non-septic ICU  miR-26b-5p and miR-193-3p: downregulated in non-septic ICU patients compared to septic ICU patients but no difference between septic ICU patients and HCs | Digital droplet PCR | “Housekeeper-independent Droplet Digital PCR technology … following the manufacturer ́s instructions” | (10) |
| High throughput sequencing: 54 altered miRNAs  miR-206 validated | Serum | 63 patients with sepsis  30 patients with septic shock  28 HCs | 29 miRNAs downregulated and 25 miRNAs upregulated in sepsis (see manuscript for sequencing results)  miR-206 upregulated in sepsis and further upregulated in septic shock | Sequencing and RT-qPCR validation | Unable to find reference gene information | (11) |
| 25 altered miRNAs in small RNA sequencing (see manuscript)  miR-1246 (validated) | Cell free miRNAs from blood (extracellular vesicles) | 30 patients with community acquired pneumonia  65 with sepsis  47 HCs | miR-1246 downregulated in sepsis compared to community acquired pneumonia and HCs (validated)  See manuscript for non-validated sequencing results | Validation by RT-qPCR | miR-30d-5p | (12) |
| miR-590-3p | Serum | 22 patients with sepsis  22 HCs | Downregulated | RT-qPCR | U6 | (13) |
| miR-146b | Plasma | 104 sepsis patients  100 HCs | Downregulated in sepsis and further downregulated in ARDS with sepsis | RT-qPCR | U6 | (14) |
| miR-125a | Plasma | 102 sepsis patients  100 HCs | Downregulated | RT-qPCR | U6 | (15) |
| miR-133a | Serum | 30 sepsis patients  30 HCs | Upregulated | RT-qPCR | U6 | (16) |
| miR-29b-3p | Plasma | 8 patients with sepsis  8 HCs | Downregulated | Data downloaded from ArrayExpre-ss repositories | Rank product analysis done (see manuscript for details) | (17) |
| miR-96-5p | Serum | 30 neonatal sepsis patients  24 respiratory infection/pneumonia patients as controls | Downregulated | RT-qPCR | U6 | (18) |
| miR-328 | Serum | 110 patients with sepsis  89 HCs | Upregulated | RT-qPCR | U6 | (19) |
| miR-103  miR-107 | Plasma | 196 sepsis patients (155 non-ARDS; 41 ARDS)  196 HCs | Downregulated in sepsis and further downregulated in sepsis plus ARDS | RT-qPCR | U6 | (20) |
| miR-126 | Plasma | 208 patients with sepsis  210 HCs | Upregulated in sepsis; further upregulated in non-survivors compared to survivors | RT-qPCR | U6 | (21) |
| miR-204-5p | Serum | 56 patients with sepsis  56 HCs | Downregulated | RT-qPCR | U6 | (22) |
| miR-146a/b | Plasma | 180 patients with sepsis  180 HCs | Upregulated | RT-qPCR | U6 | (23) |
| miR-103 | Serum | 108 sepsis  89 non-infectious SIRS  68 HCs | Downregulated in sepsis and non-infectious SIRS compared to HCs, sepsis patients downregulated further compared to SIRS | RT-qPCR | U6 | (24) |
| miR-103a-3p | Serum | 30 sepsis patients  30 HCs | Upregulated | RT-qPCR | U6 | (25) |
| miR-150 | Serum | 41 sepsis patients | Downregulated in non-survivors compared to survivors | RT-qPCR | Exogenous Cel-miR-39-3p | (26) |
| miR-125a | Plasma | 196 patients with sepsis  196 HCs | downregulated | RT-qPCR | U6 | (27) |
| miR-19b-3p | Serum | 103 patients with sepsis  98 HCs | downregulated | RT-qPCR | U6 | (28) |
| miR-181a (synonymous to miR-181a-5p) | Serum | 102 neonates with sepsis  50 healthy neonates | downregulated | RT-qPCR | U6 | (29) |
| miR-125a and miR-125b | Plasma | 150 patients with sepsis  150 HCs | miR125a: upregulated  miR-125b: upregulated in sepsis compared to HCs; upregulated in non-survivors compared to survivors | RT-qPCR | U6 | (30) |
| miR-21 | Plasma | 217 patients with sepsis  217 HCs | Downregulated | RT-qPCR | U6 | (31) |
| miR-125a and miR-125b | Plasma | 120 patients with sepsis  120 HCs | miR125a: no changes with disease  miR-125b: upregulated in sepsis compared to HCs; decreased in survivors compared to non-survivors | RT-qPCR | U6 | (32) |
| miR-181a-5p | Whole blood | 136 septic patients  151 HCs | Downregulated | RT-qPCR | U6 | (33) |
| miR-130b-3p | Serum | 15 septic patients,  7 HCs | Upregulated | RT-qPCR | Spiked in C. elegans miRNA 39-3p | (34) |
| miR-3158-3p  miR-193a-3p  miR-144-3p  miR-4685-3p  miR-202-5p  miR-142-5p  miR-625-3p  miR-106b-3p  miR-106b-5p  miR-128-3p  miR-144-5p  miR-15b-3p  miR-2110  miR-181a-5p  miR-223-3p  miR-378a-3p  miR-143-3p  miR-320a  miR-423-5p  let-7b-5p  miR-320b  miR-34a-5p  miR-4732-5p | Whole blood | 3 septic patients  3 HCs | Downregulated:  miR-3158-3p  miR-193a-3p  miR-144-3p  miR-4685-3p  miR-202-5p  miR-142-5p  miR-625-3p  miR-106b-3p  miR-106b-5p  miR-128-3p  miR-144-5p  miR-15b-3p  Upregulated  miR-2110  miR-181a-5p  miR-223-3p  miR-378a-3p  miR-143-3p  miR-320a  miR-423-5p  let-7b-5p  miR-320b  miR-34a-5p  miR-4732-5p | Sequencing | ---- | (35) |
| miR-145 | Exosomes | 33 sepsis patients  32 healthy volunteers | Downregulated | RT-qPCR | U6 | (36) |
| miR-106a | Plasma  “Serum” as per authors, but actually plasma (whole blood preserved by anticoagulation then centrifuged) | 50 sepsis patients  30 HCs | Upregulated | RT-qPCR | Not stated | (37) |
| miR-155 | Serum | 218 patients with critical illness (non-sepsis, sepsis, septic shock)  76 HCs | Upregulated in critically ill patients (approximately 10 fold, not specific to sepsis)  significant lower levels of miR-155 in patients < age 65; within age group < 65, lower miR-155 levels correlated significantly with mortality outcome | RT-qPCR | Spiked in miRNA mimic SV40 | (38) |
| miR-494-3p | Plasma | Sepsis versus HCs (patient numbers not specified) | Downregulated | RT-qPCR | U6 | (39) |
| miR-223-3p  miR-7110-5p | Plasma | 52 patients with pneumonia, 44 patients with sepsis secondary to pneumonia and 21 HCs | Both miRNAs upregulated in both patient groups compared to HCs; miR-223-3p approx. 2 fold greater in sepsis compared to pneumonia alone | RT-qPCR | miR-16 | (40) |
| miR-25 | Whole blood collected in PAXgene RNA tubes | 216 newborns with sepsis and 242 newborns without sepsis | Downregulated in septic newborns | RT-qPCR | U6 | (41) |
| miR-187  miR-21  miR-145 | Plasma | 102 patients with mild sepsis; 78 patients with severe sepsis; 180 HCs | Downregulated: miR-187  Upregulated:  miR-21 and miR-145 | RT-qPCR | U6 | (42) |
| miR-122 | Serum | 108 patients with sepsis; 20 controls with infection but no sepsis | Upregulated | RT-qPCR | Spiked in cel-miR-54 | (43) |
| miR-142 | Total RNA (although not explicitly stated) | 31 patients with sepsis  22 healthy volunteers | Downregulated in sepsis | RT-qPCR | U6 | (44) |
| miR-7-5p | Exosomes | 6 septic patients  6 HCs | Upregulated in sepsis | RT-qPCR | U6 | (45) |
| miR-124 | Plasma | 82 septic patients  82 HCs | Decreased in sepsis | RT-qPCR | U6 | (46) |
| miR-146a | Serum | 55 septic children  60 healthy children | Decreased in sepsis and correlated with severity: lowest in septic shock than in severe sepsis and sepsis. Also lower in non-survivors compared to survivors | RT-qPCR | U6 | (47) |
| miR-122  miR-133a  miR-143  miR-150  miR-155  miR-223 | Serum | 127 septic patients; 77 non-sepsis critical illness; HCs (number not specified) | MiRNAs upregulated in critical illness compared to HCs: miR-122; miR-133a;  miR-155 and miR-192.  miRNAs lower in patients compared to HCs: miR-150 and miR-223.  Upregulated in sepsis compared to non-septic critical illness: miR-133a.  In non-survivors compared to survivors: miR-133a was elevated, miR-143 and miR-223 were decreased.  Unfavorable prognosis: miR-133a (upregulated) and miR-150 (downregulated) | RT-qPCR | Spiked in miRNA mimic SV40 | (48) |
| miR-21 | Whole blood | 88 children septic shock  26 HCs | Upregulated in septic shock | Genome wide expression profiling | -- | (49) |
| miR-874 | Plasma | 69 sepsis patients  57 HCs | Upregulated | RT-qPCR | U6 | (50) |
| miR-150 | Plasma | 120 patients with sepsis  50 HCs | Downregulated | RT-qPCR | U6 | (51) |
| miR-150  miR-342  miR-15a  miR-16  miR-93  miR-143  miR-223  miR-424 | T cells (one cohort)  Whole blood (separate cohort) | T cell analysis: 30 patients with sepsis; unclear number of HCs  Whole blood: 20 patients with sepsis and 10 HCs | Downregulated in septic T cells: miR-150 and miR-342;  Upregulated in septic T cells: miR-15a, miR-16, miR-93, miR-143, miR-223 and miR-424.  Downregulated in whole blood: miR-150, miR-342, miR-16, miR-93  Upregulated in whole blood: miR-143 | RT-qPCR | U47 | (52) |
| 86 miRNAs | Exosomes | 24 patients with septic shock on day 1 of admission and 7 days later  12 healthy volunteers | 65 differentially expressed miRNAs between sepsis day 1 and HCs and sepsis day 7 versus HCs. 35 differentially expressed miRNAs between sepsis survivors versus non-survivors.  Refer to supplementary materials of paper for details (tables S1 and S2).  MiRNAs that had fold changes greater than or equal to 10 (fold changes in brackets):  Day 1 vs. HCs:  miR-122-5p (13.6)  miR-1262 (826)  miR-1290 (25.3)  miR-1298-5p (118.4)  miR-193a-5p (11.1)  miR-885-5p (28.7)  Day 7 vs HCs:  miR-122-5p (14)  miR-1227-3p (15.1)  miR-1262 (980.4)  miR-1290 (17.9)  miR-1298-5p (167.7)  miR-1300 (23.1)  miR-18a-5p (-12.3)  miR-340-5p (-21.9)  miR-518d-3p (14.9)  miR-519b-3p (71.5)  miR-520d-3p (16.1)  miR-548a-3p (44.8)  miR-548c-3p (13.8)  miR-618 (14.3)  miR-770-5p (12.3)  miR-885-5p (28.9)  Sepsis survivors vs. non-survivors:  miR-1183 (33.9)  miR-1233-3p (55.3)  miR-1243 (-53.6)  miR-1262 (-2,288.1)  miR-29b-3p (-26.5)  miR-339-3p (12.7)  miR-520d-5p (-63.5)  miR-548a-3p (-20.4)  miR-549a (-11,616.4)  miR-598-3p (28.9)  miR-618 (-10.7)  miR-766-3p (34.6)  miR-875-5p (-18.9) | RT-qPCR | Genorm identified:  miR-17  miR-20a  miR-106a | (53) |
| miR-221  miR-222 | PBMCs from peripheral blood | 10 patients with sepsis  10 HCs | Upregulated in sepsis | RT-qPCR | Unclear normalization strategy | (54) |
| miR-155 | Plasma | 27 patients with septic shock versus ICU non-septic patients | Upregulated in sepsis  Upregulated in nonsurvivors compared to survivors | RT-qPCR | Three synthetic miRs spiked-in to plasma: cel-miR-39, cel-miR-54 and cel-miR-238 | (55) |
| miR-223 | Plasma | 187 patients with sepsis  186 HCs | Upregulated in sepsis  Upregulated in non-survivors | RT-qPCR | U6 | (56) |
| miR-378-3p  miR-30a-5p  miR-30d-5p  miR-192-5p | Plasma | Sepsis 29  Noninfective SIRS 44 | The following miRNAs had significant positive correlations with disease severity (SOFA score) in SIRS only (no significance in sepsis):  miR-378-3p  miR-30a-5p  miR-30d-5p  miR-192-5p | RT-qPCR | miR-320a and miR-486-5p  (as identified by Normfinder) | (57) |
| miR-142-5p  miR-191-5p  miR-22-3p  miR-23a-3p  miR-3165  miR-4456 | Serum | Sepsis-induced AKI (n=35)  Sepsis non-AKI (n=30)  HCs (n=20) | Significantly upregulate in both sepsis groups (AKI and non-AKI) compared to controls: miR-3165  Significantly downregulated in both sepsis groups (AKI and non-AKI) compared to controls:  miR-142-5p  miR-22-3p  miR-191-5p  miR-23a-3p  miR-4456 | RT-qPCR | miR-423-5p | (58) |
| miR-107 | Circulating endothelial cells | 5 patients each:  Septic AKI  Non-septic AKI  Septic non-AKI  HCs | miR-107 increased in septic AKI group compared to the rest of the groups. | RT-qPCR | U6 | (59) |
| miR-135a | Serum | 69 patients with sepsis  53 HCs | Upregulated in sepsis compared to HCs  Significantly upregulated in severe sepsis compared to moderate sepsis | RT-qPCR | U6 | (60) |
| miR-375  miR-21 | Whole blood | 33 “late-stage”sepsis patients  18 HCs | miR-375 downregulated  miR-21 upregulated | RT-qPCR | U6 | (61) |
| miR-30d  miR-30a  miR-192  miR-26a  miR-23a  miR-191 | Plasma | 21 patients with sepsis  23 patients with SIRS | All downregulated in sepsis compared to SIRS | RT-qPCR | miR-320a and miR-486-5p (identified by Normfinder) | (62) |
| miR-143 | Serum | 103 patients with sepsis  95 patients with SIRS  40 HCs | Upregulated in SIRS and sepsis compared to controls  Upregulated in sepsis compared to SIRS | RT-qPCR | U6 | (63) |
| miR-125b | PBMCs | “infected patients” (not clearly septic by definition) n=6  HCs (n=6) | Downregulated in sepsis | RT-qPCR | U6 | (64) |
| miR-223 | Serum | 157 patients with sepsis  84 patients with non-septic critical illness | No difference between septic patients and non-septic critical illness or HCs;  Downregulated in critically ill patients with high APACHE score | RT-qPCR | Spiked in SV40 | (65) |
| miR-19a | B cells from blood | 38 sepsis patients  26 non infected SIRS  15 healthy volunteers | Upregulated in sepsis compared to non-infective SIRS  Upregulated in non-infective SIRS and sepsis compared to HCs | RT-qPCR | U6 | (66) |
| miR-15a  miR-27a  miR-34a  miR-126  miR-21  miR-16  miR-150  miR-223  miR-181b  miR-146a  miR-155  miR-125b  miR-486 | Plasma | 62 septic patients (29 in shock and 33 without shock)  32 HCs | Upregulated in sepsis patients compared to HCs: all miRNAs (except miR-150 and miR-486)  Downregulated in septic shock versus sepsis: miR-15a and miR-27a  Upregulated in septic shock: miR-34a  Downregulated in patients with AKI: miR-15a | RT-qPCR | RNU6B | (67) |
| Let-7a and miR-150 | Peripheral leukocytes | 22 gram negative urosepsis patients  20 HCs | Both downregulated in sepsis | RT-qPCR | U6 | (68) |
| miR-155 | Whole blood | 60 patients with sepsis  30 HCs | Upregulated | RT-qPCR | Cel-miR-39 | (69) |
| miR-146a | T cells | 16 septic patients  16 HCs | Downregulated | RT-qPCR | U47 | (70) |
| miR-182  miR-143  miR-145  miR-146a  miR-150  miR-155 | PBMCs | Sepsis patients day 1 post admission  HCs  (For miRNA array analysis, sepsis n=2; HC = 4  For RT-qPCR, 12 sepsis and 12 HCs) | Upregulated in array analysis:  miR-182  miR-143  miR-486  miR-145  miR-1308  Downregulated in array analysis:  miR-181  miR-146a  miR-584  miR-155  Confirmed with RT-qPCR:  miR-182  miR-486  miR-146a | Affymetrix miRNA array analysis  RT-qPCR | RNU1A | (71) |
| miR-29a  miR-96  miR-141  miR-181a  miR- 1184  miR-101  miR-185 | Peripheral blood leukocytes | 24 neonates with sepsis at the time of diagnosis (12 with gram +ve and 12 with gram -ve)  24 uninfected neonates as controls upon admission to neonatal unit | Confirmed by RT-qPCR, downregulated in sepsis:  miR-29a  miR-96  miR-141  miR-181a  miR-1184  Upregulated in sepsis:  miR-101  miR-185 | miRCURY LNA miRNA array analysis  Validation with RT-qPCR | Reference genes not specified | (72) |
| miR-133a | Serum | 223 critically ill patients (138 with sepsis, 85 without sepsis)  76 HCs  Analysis done at ICU admission and day 3 | miR-133a elevated on ICU admission in critically ill patients and more so in septic patients (compared to HCs)  Levels correlated with prognosis/survival | RT-qPCR | Spiked in SV40 | (73) |
| Numerous miRNAs (see original paper)  NB: in this study they show that different etiologies of sepsis affect miRNAs differently | Mononuclear cells (primary leukocytes)  Plasma miRNAs in two different cohorts from separate centres in different countries “FCH” and “MDACC” | Mononuclear cells: 8 septic and 8 HCs  FCH: 33 sepsis patients and 53 HCs  MDACC: 66 sepsis and 53 HCs | miR-16, miR-182 and miR-486 were overexpressed;  miR-23a, miR-26a, miR-26b, miR-93, miR-146a, miR-150, miR-342-3p and miR-342-5p were downregulated in MNCs from septic patients compared with healthy humans  Upregulated in sepsis in FCH cohort (confirmed by RT-qPCR): none  Downregulated in FCH cohort: miR-16-5p, miR-23a-3p, miR-26a-5p, miR-93-5p, miR-182-5p, miR-342-3p, miR-486-5p  MDACC cohort (RT-qPCT confirmed) upregulated in sepsis: miR-16-5p, 23a-3p, 26a-5p, 26b-5p, 93-5p, 182-5p, 342-3p, 486-5p | miRNAomic microarray analysis  RT-qPCR | Spiked in cel-39-3p; U6 as housekeeping | (74) |
| miR-122  miR-483-5p  miR-574-5p | Serum | 232 sepsis  24 HCs | Downregulated: miR-122, miR-483-5p, miR-574-5p | RT-qPCR | 5S rRNA | (75) |
| miR-146a | Plasma | Two cohorts, each with sepsis patients and  non-sepsis SIRS as controls  Cohort 1: sepsis n=4, non-sepsis SIRS n=10  Cohort 2: sepsis n=6, non-sepsis SIRS n=8 | miR-146a decreased in expression in sepsis compared to non-sepsis SIRS in both cohorts | RT-qPCR | U6 (but also demonstrated that it does not vary in their samples) | (76) |
| Novel miRNAs | Serum | RT-qPCR validation on 32 sepsis survivors,  24 nonsurvivors, 14 HCs | Differentially expressed miRNA (total 6) all novel and unannotated | Solexa sequencing  RT-qPCR | 5S rRNA | (77) |
| miR-146a | PBMCs | 37 patients with severe sepsis    40 HCs | miR-146a downregulated in severe sepsis | RT-qPCR | U6 | (78) |
| U6 | Serum | 203 critically ill patients on admission to ICU (126 had bacterial sepsis, all grouped together)  HCs n=44 | Upregulated in critical illness | RT-qPCR | Compared to spiked in SV40 RNA | (79) |
| miR-4661 | Whole blood  Plasma | E. coli sepsis survivors (n=3)  E. coli sepsis non-survivors (n=3)  Nonseptic ICU control patients (n=3)  Blood taken within 48 hrs of admission | Significantly upregulated in whole blood in sepsis, also further upregulated in non-survivors compared to survivors  In plasma, unregulated significantly in non-survivors. | RT-qPCR | Spiked in cel-miR-39 | (80) |
| miR-150  miR-4772-5p-iso | Whole blood | HCs (n=17)  Sepsis (n=22)  SIRS (n=22) | Upregulated in sepsis compared to HC and SIRS: miR-342-3p,  miR-3173-5p and miR-150 (miR-150 is also upregulated in SIRS compared to HCs)  Upregulated in sepsis compared to HCs: miR-191 iso  Downregulated in sepsis and SIRS compared to HCs (no difference between sepsis and SIRS): miR-4772-3p and miR-4772-5p  Downregulated in sepsis compared to HCs and SIRS: miR-4772-5p-iso | RT-qPCR | Raw CT values used due to lack of consensus on normalization of miRNA | (81) |
| miR-150 | Serum | 138 patients with sepsis  85 critically unwell without sepsis  76 HCs | Levels of miR-150 unchanged in septic patients  No variation between different etiologies of sepsis  Low serum miR-150 predicted mortality, correlated significantly with hepatic or renal dysfunction | RT-qPCR | Spiked in miRNA mimic SV40 | (82) |
| miR-297  miR-574-5p | Serum | Microarray: 12 surviving and 12 non-surviving sepsis patients  RT-qPCR validation: 118 patients with sepsis (66 survivors, 52 non-survivors) | miR-297 upregulated in non-survivors  miR-574-5p downregulated in non-survivors | Microarray and RT-qPCR | 5S rRNA as control for RT-qPCR | (83) |
| miR-15  miR-16 | Peripheral leukocytes | Cirrhotic patients with bacterial infection n=19  Cirrhotic patients without infection n=20  HCs n=5 | miR-15 and miR-16 both upregulated in patients (cirrhotics, no infection, cirrhotic with infection) compared to HCs | RT-qPCR | Unclear internal control 🡪 seemingly 5S rRNA | (84) |
| miR-181b | plasma | Patients with sepsis (n=26)  Patients with sepsis plus ARDS (n=36)  Control patients admitted to ICU without sepsis (n=16) | Downregulated in sepsis compared to controls  Downregulated in sepsis/ARDS compared to controls | RT-qPCR | Unclear but seemingly RNU6B | (85) |
| miR-15a  miR-16 | Serum | Sepsis (n=166)  SIRS (n=32)  HCs (n=24) | miR-15a higher in sepsis and SIRS compared to controls  miR-15a higher in SIRS compared to sepsis  No difference in miR-16 between SIRS and sepsis  miR-16 higher in SIRS and sepsis compared to controls | RT-qPCR | U6 | (86) |
| miR-15b  miR-223  miR-122  miR-193b  miR-483-5p  miR-499-5p | Serum | 43 mild sepsis  73 (severe sepsis and septic shock)  24 HCs | miR-15b: upregulated in sepsis compared to HCs; downregulated in severe sepsis/septic shock compared to sepsis  miR-122: downregulated in sepsis compared to HCs; downregulated in severe sepsis/septic shock compared to HCs  miR-223: upregulated in sepsis compared to HCs; upregulated in severe sepsis/septic shock compared to HCs; downregulated in severe sepsis/septic shock compared to sepsis  miR-193b: downregulated in sepsis compared to HCs; downregulated in severe sepsis/septic shock compared to HCs  miR-483-5p: upregulated in sepsis compared to HCs; downregulated in severe sepsis/septic shock compared to sepsis  miR-499-5p: downregulated in severe sepsis/septic shock compared to sepsis and compared to HCs | RT-qPCR | U6 | (87) |
| miR-15a  miR-16  miR-122  miR-193b*  miR-223  miR-483-5p  miR-499-5p | Serum | Sepsis survivors (n=15) compared to non-survivors (n=15) | Upregulated in non-survivors: miR-15a, miR-483-5p, miR-122, miR-193b*, miR-499-5p  Downregulated in non-survivors: miR-16, miR-223 | RT-qPCR | U6 | (88) |
| miR-146a  miR-223  miR-126  miR-15b  miR-132  miR-155  let-7i | Serum | 50 patients with sepsis  30 patients with SIRS  20 HCs | miR-146a and miR-223: decreased in septic patients compared to SIRS and normal controls  miR-146a: reduced in SIRS compared to normal controls  miR-223: no difference between SIRS and normal controls  miR-126: reduced in sepsis and SIRS compared to normal, but no difference between sepsis and SIRS  miR-132, miR-15b and miR-155: similar across all groups | RT-qPCR | Spiked in mmu-miR-295 | (89) |
| miR-146b  miR-150  miR-342  let-7g  miR-143 | Leukocyte RNA | 3 healthy volunteers injected with LPS as a model of early sepsis pathogenesis for microarray  N=6 healthy patients before and after LPS for RT-qPCR validation | Microarray data  Downregulated after LPS: miR-146b, miR-150, miR-342, let-7g  Upregulated after LPS: miR-143  RT-qPCR used to verify 3 miRNAs: let-7g, miR-150, miR-342; downregulation confirmed for miR-150 and miR-342 but not let-7g | Microarray and RT-qPCR | U6 | (90) |
| miR-150  miR-182  miR-342-5p  miR-486 | Peripheral blood leukocytes (microarray screening)  Plasma | 8 sepsis patients on day 1 and 8 HCs for microarray  RT-qPCR:  24 sepsis (16 at day 1, 8 at day 7); 32 HCs | Microarray leukocytes:  Upregulated in sepsis: miR-486 and miR-182  Downregulated in sepsis: miR-150 and miR-342-5p  RT-qPCR on same microarray RNA (leukocytes) confirmed miR-182, miR-150 and miR-342-5p patterns | RT-qPCR | miR-192 (state that U6B in plasma was too unstable)  for leukocyte experiments U6B | (91) |

**References:**

1. Gao M, Yu T, Liu D, Shi Y, Yang P, Zhang J, et al. Sepsis plasma-derived exosomal miR-1-3p induces endothelial cell dysfunction by targeting SERP1. Clin Sci (Lond). 2021;135(2):347-65.

2. Scicluna BP, Uhel F, van Vught LA, Wiewel MA, Hoogendijk AJ, Baessman I, et al. The leukocyte non-coding RNA landscape in critically ill patients with sepsis. Elife. 2020;9.

3. Liu Z, Yang D, Gao J, Xiang X, Hu X, Li S, et al. Discovery and validation of miR-452 as an effective biomarker for acute kidney injury in sepsis. Theranostics. 2020;10(26):11963-75.

4. Ahmad S, Ahmed MM, Hasan PMZ, Sharma A, Bilgrami AL, Manda K, et al. Identification and Validation of Potential miRNAs, as Biomarkers for Sepsis and Associated Lung Injury: A Network-Based Approach. Genes (Basel). 2020;11(11).

5. Yang W, Luo X, Liu Y, Xiong J, Xia H, Liu Y. Potential role of lncRNA HULC/miR1283p/RAC1 axis in the inflammatory response during LPSinduced sepsis in HMEC1 cells. Mol Med Rep. 2020;22(6):5095-104.

6. Liu D, Wang Z, Wang H, Ren F, Li Y, Zou S, et al. The protective role of miR-223 in sepsis-induced mortality. Sci Rep. 2020;10(1):17691.

7. Heng J, Wu D, Lu S, Zhao Y. miR-106a Targets Anoctamin 1 (ANO1) to Regulate Lipopolysaccharide (LPS)-Induced Inflammatory Response in Macrophages. Med Sci Monit. 2020;26:e922479.

8. Fatmi A, Rebiahi SA, Chabni N, Zerrouki H, Azzaoui H, Elhabiri Y, et al. miRNA-23b as a biomarker of culture-positive neonatal sepsis. Mol Med. 2020;26(1):94.

9. Wei B, Yu L. Circular RNA PRKCI and microRNA-545 relate to sepsis risk, disease severity and 28-day mortality. Scand J Clin Lab Invest. 2020;80(8):659-66.

10. Link F, Krohn K, Burgdorff AM, Christel A, Schumann J. Sepsis Diagnostics: Intensive Care Scoring Systems Superior to MicroRNA Biomarker Testing. Diagnostics (Basel). 2020;10(9).

11. Liang G, Wu Y, Guan Y, Dong Y, Jiang L, Mao G, et al. The correlations between the serum expression of miR-206 and the severity and prognosis of sepsis. Ann Palliat Med. 2020;9(5):3222-34.

12. Hermann S, Brandes F, Kirchner B, Buschmann D, Borrmann M, Klein M, et al. Diagnostic potential of circulating cell-free microRNAs for community-acquired pneumonia and pneumonia-related sepsis. J Cell Mol Med. 2020;24(20):12054-64.

13. Liu L, Liu F, Sun Z, Peng Z, You T, Yu Z. LncRNA NEAT1 promotes apoptosis and inflammation in LPS-induced sepsis models by targeting miR-590-3p. Exp Ther Med. 2020;20(4):3290-300.

14. Chen W, Liu L, Yang J, Wang Y. MicroRNA-146b correlates with decreased acute respiratory distress syndrome risk, reduced disease severity, and lower 28-day mortality in sepsis patients. J Clin Lab Anal. 2020;34(12):e23510.

15. Yang Y, Yang L, Liu Z, Wang Y, Yang J. Long noncoding RNA NEAT 1 and its target microRNA-125a in sepsis: Correlation with acute respiratory distress syndrome risk, biochemical indexes, disease severity, and 28-day mortality. J Clin Lab Anal. 2020;34(12):e23509.

16. Chen L, Xie W, Wang L, Zhang X, Liu E, Kou Q. MiRNA-133a aggravates inflammatory responses in sepsis by targeting SIRT1. Int Immunopharmacol. 2020;88:106848.

17. Li Z, Yi N, Chen R, Meng Y, Wang Y, Liu H, et al. miR-29b-3p protects cardiomyocytes against endotoxin-induced apoptosis and inflammatory response through targeting FOXO3A. Cell Signal. 2020;74:109716.

18. Chen X, Chen Y, Dai L, Wang N. MiR-96-5p alleviates inflammatory responses by targeting NAMPT and regulating the NF-kappaB pathway in neonatal sepsis. Biosci Rep. 2020;40(7).

19. Sun B, Luan C, Guo L, Zhang B, Liu Y. Low expression of microRNA-328 can predict sepsis and alleviate sepsis-induced cardiac dysfunction and inflammatory response. Braz J Med Biol Res. 2020;53(8):e9501.

20. Wang Q, Feng Q, Zhang Y, Zhou S, Chen H. Decreased microRNA 103 and microRNA 107 predict increased risks of acute respiratory distress syndrome and 28-day mortality in sepsis patients. Medicine (Baltimore). 2020;99(25):e20729.

21. Lin R, Hu H, Li L, Chen G, Luo L, Rao P. The potential of microRNA-126 in predicting disease risk, mortality of sepsis, and its correlation with inflammation and sepsis severity. J Clin Lab Anal. 2020;34(9):e23408.

22. Chen X, Song D. LPS promotes the progression of sepsis by activation of lncRNA HULC/miR-204-5p/TRPM7 network in HUVECs. Biosci Rep. 2020;40(6).

23. Chen L, Yu L, Zhang R, Zhu L, Shen W. Correlation of microRNA-146a/b with disease risk, biochemical indices, inflammatory cytokines, overall disease severity, and prognosis of sepsis. Medicine (Baltimore). 2020;99(22):e19754.

24. Yang M, Zhao L, Sun M. Diagnostic Value of miR-103 in Patients with Sepsis and Noninfectious SIRS and Its Regulatory Role in LPS-Induced Inflammatory Response by Targeting TLR4. Int J Genomics. 2020;2020:2198308.

25. Zhou YP, Xia Q. Inhibition of miR-103a-3p suppresses lipopolysaccharide-induced sepsis and liver injury by regulating FBXW7 expression. Cell Biol Int. 2020;44(9):1798-810.

26. Braza-Boils A, Barwari T, Gutmann C, Thomas MR, Judge HM, Joshi A, et al. Circulating MicroRNA Levels Indicate Platelet and Leukocyte Activation in Endotoxemia Despite Platelet P2Y12 Inhibition. Int J Mol Sci. 2020;21(8).

27. Liu W, Geng F, Yu L. Long non-coding RNA MALAT1/microRNA 125a axis presents excellent value in discriminating sepsis patients and exhibits positive association with general disease severity, organ injury, inflammation level, and mortality in sepsis patients. J Clin Lab Anal. 2020;34(6):e23222.

28. Xu H, Liu X, Ni H. Clinical significance of miR-19b-3p in patients with sepsis and its regulatory role in the LPS-induced inflammatory response. Eur J Med Res. 2020;25(1):9.

29. Liu G, Liu W, Guo J. Clinical significance of miR-181a in patients with neonatal sepsis and its regulatory role in the lipopolysaccharide-induced inflammatory response. Exp Ther Med. 2020;19(3):1977-83.

30. Li S, Zhao D, Cui J, Wang L, Ma X, Li Y. Correlation of microRNA-125a/b with acute respiratory distress syndrome risk and prognosis in sepsis patients. J Clin Lab Anal. 2020;34(3):e23098.

31. Na L, Ding H, Xing E, Zhang Y, Gao J, Liu B, et al. The predictive value of microRNA-21 for sepsis risk and its correlation with disease severity, systemic inflammation, and 28-day mortality in sepsis patients. J Clin Lab Anal. 2020;34(3):e23103.

32. Zhu X. MiR-125b but not miR-125a is upregulated and exhibits a trend to correlate with enhanced disease severity, inflammation, and increased mortality in sepsis patients. J Clin Lab Anal. 2020;34(3):e23094.

33. Wang Y, Xu Z, Yue D, Zeng Z, Yuan W, Xu K. Linkage of lncRNA CRNDE sponging miR-181a-5p with aggravated inflammation underlying sepsis. Innate Immun. 2020;26(2):152-61.

34. Gurien SD, Aziz M, Jin H, Wang H, He M, Al-Abed Y, et al. Extracellular microRNA 130b-3p inhibits eCIRP-induced inflammation. EMBO Rep. 2020;21(1):e48075.

35. Qin Y, Guo X, Yu Y, Dong S, Yan Y, Bian X, et al. Screening key genes and microRNAs in sepsis by RNA-sequencing. J Chin Med Assoc. 2020;83(1):41-7.

36. Cao X, Zhang C, Zhang X, Chen Y, Zhang H. MiR-145 negatively regulates TGFBR2 signaling responsible for sepsis-induced acute lung injury. Biomed Pharmacother. 2019;111:852-8.

37. Shen Y, Yu J, Jing Y, Zhang J. MiR-106a aggravates sepsis-induced acute kidney injury by targeting THBS2 in mice model. Acta Cir Bras. 2019;34(6):e201900602.

38. Tacke F, Spehlmann ME, Vucur M, Benz F, Luedde M, Cardenas DV, et al. miR-155 Predicts Long-Term Mortality in Critically Ill Patients Younger than 65 Years. Mediators Inflamm. 2019;2019:6714080.

39. Wang HF, Li Y, Wang YQ, Li HJ, Dou L. MicroRNA-494-3p alleviates inflammatory response in sepsis by targeting TLR6. Eur Rev Med Pharmacol Sci. 2019;23(7):2971-7.

40. Zhang W, Jia J, Liu Z, Si D, Ma L, Zhang G. Circulating microRNAs as biomarkers for Sepsis secondary to pneumonia diagnosed via Sepsis 3.0. BMC Pulm Med. 2019;19(1):93.

41. Zheng G, Xiang W, Pan M, Huang Y, Li Z. Identification of the association between rs41274221 polymorphism in the seed sequence of microRNA-25 and the risk of neonate sepsis. J Cell Physiol. 2019.

42. Zhang Y, Li M, Bao L, Hu P. A case-control study on the relationship between miRNAs single nucleotide polymorphisms and sepsis risk. Medicine (Baltimore). 2019;98(33):e16744.

43. Rahmel T, Schafer ST, Frey UH, Adamzik M, Peters J. Increased circulating microRNA-122 is a biomarker for discrimination and risk stratification in patients defined by sepsis-3 criteria. PLoS One. 2018;13(5):e0197637.

44. Zhen J, Chen W. MiR-142 inhibits cecal ligation and puncture (CLP)-induced inflammation via inhibiting PD-L1 expression in macrophages and improves survival in septic mice. Biomed Pharmacother. 2018;97:1479-85.

45. Deng JN, Li YQ, Liu Y, Li Q, Hu Y, Xu JQ, et al. Exosomes derived from plasma of septic patients inhibit apoptosis of T lymphocytes by down-regulating bad via hsa-miR-7-5p. Biochem Biophys Res Commun. 2019;513(4):958-66.

46. He F, Zhang C, Huang Q. Long noncoding RNA nuclear enriched abundant transcript 1/miRNA-124 axis correlates with increased disease risk, elevated inflammation, deteriorative disease condition, and predicts decreased survival of sepsis. Medicine (Baltimore). 2019;98(32):e16470.

47. Karam RA, Zidan HE, Karam NA, Abdel Rahman DM, El-Seifi OS. Diagnostic and prognostic significance of serum miRNA-146-a expression in Egyptian children with sepsis in a pediatric intensive care unit. J Gene Med. 2019;21(11):e3128.

48. Roderburg C, Benz F, Koch A, Loosen SH, Spehlmann M, Luedde M, et al. A Combined Score of Circulating miRNAs Allows Outcome Prediction in Critically Ill Patients. J Clin Med. 2019;8(10).

49. Xue Z, Xi Q, Liu H, Guo X, Zhang J, Zhang Z, et al. miR-21 promotes NLRP3 inflammasome activation to mediate pyroptosis and endotoxic shock. Cell Death Dis. 2019;10(6):461.

50. Fang Y, Hu J, Wang Z, Zong H, Zhang L, Zhang R, et al. LncRNA H19 functions as an Aquaporin 1 competitive endogenous RNA to regulate microRNA-874 expression in LPS sepsis. Biomed Pharmacother. 2018;105:1183-91.

51. Ma Y, Liu Y, Hou H, Yao Y, Meng H. MiR-150 predicts survival in patients with sepsis and inhibits LPS-induced inflammatory factors and apoptosis by targeting NF-kappaB1 in human umbilical vein endothelial cells. Biochem Biophys Res Commun. 2018;500(3):828-37.

52. Mohnle P, Hirschberger S, Hinske LC, Briegel J, Hubner M, Weis S, et al. MicroRNAs 143 and 150 in whole blood enable detection of T-cell immunoparalysis in sepsis. Mol Med. 2018;24(1):54.

53. Real JM, Ferreira LRP, Esteves GH, Koyama FC, Dias MVS, Bezerra-Neto JE, et al. Exosomes from patients with septic shock convey miRNAs related to inflammation and cell cycle regulation: new signaling pathways in sepsis? Crit Care. 2018;22(1):68.

54. Seeley JJ, Baker RG, Mohamed G, Bruns T, Hayden MS, Deshmukh SD, et al. Induction of innate immune memory via microRNA targeting of chromatin remodelling factors. Nature. 2018;559(7712):114-9.

55. Vasques-Novoa F, Laundos TL, Cerqueira RJ, Quina-Rodrigues C, Soares-Dos-Reis R, Baganha F, et al. MicroRNA-155 Amplifies Nitric Oxide/cGMP Signaling and Impairs Vascular Angiotensin II Reactivity in Septic Shock. Crit Care Med. 2018;46(9):e945-e54.

56. Wu X, Yang J, Yu L, Long D. Plasma miRNA-223 correlates with risk, inflammatory markers as well as prognosis in sepsis patients. Medicine (Baltimore). 2018;97(27):e11352.

57. Caserta S, Mengozzi M, Kern F, Newbury SF, Ghezzi P, Llewelyn MJ. Severity of Systemic Inflammatory Response Syndrome Affects the Blood Levels of Circulating Inflammatory-Relevant MicroRNAs. Front Immunol. 2017;8:1977.

58. Ge QM, Huang CM, Zhu XY, Bian F, Pan SM. Differentially expressed miRNAs in sepsis-induced acute kidney injury target oxidative stress and mitochondrial dysfunction pathways. PLoS One. 2017;12(3):e0173292.

59. Wang S, Zhang Z, Wang J, Miao H. MiR-107 induces TNF-alpha secretion in endothelial cells causing tubular cell injury in patients with septic acute kidney injury. Biochem Biophys Res Commun. 2017;483(1):45-51.

60. Zheng G, Pan M, Jin W, Jin G, Huang Y. MicroRNA-135a is up-regulated and aggravates myocardial depression in sepsis via regulating p38 MAPK/NF-kappaB pathway. Int Immunopharmacol. 2017;45:6-12.

61. Sheng B, Zhao L, Zang X, Zhen J, Chen W. miR-375 ameliorates sepsis by downregulating miR-21 level via inhibiting JAK2-STAT3 signaling. Biomed Pharmacother. 2017;86:254-61.

62. Caserta S, Kern F, Cohen J, Drage S, Newbury SF, Llewelyn MJ. Circulating Plasma microRNAs can differentiate Human Sepsis and Systemic Inflammatory Response Syndrome (SIRS). Sci Rep. 2016;6:28006.

63. Han Y, Dai QC, Shen HL, Zhang XW. Diagnostic value of elevated serum miRNA-143 levels in sepsis. J Int Med Res. 2016;44(4):875-81.

64. Zhang F, Fan X, Bai Y, Lu J, Zheng M, Chen J, et al. miR-125b regulates procalcitonin production in monocytes by targeting Stat3. Microbes Infect. 2016;18(2):102-8.

65. Benz F, Tacke F, Luedde M, Trautwein C, Luedde T, Koch A, et al. Circulating microRNA-223 serum levels do not predict sepsis or survival in patients with critical illness. Dis Markers. 2015;2015:384208.

66. Jiang Y, Zhou H, Ma D, Chen ZK, Cai X. MicroRNA-19a and CD22 Comprise a Feedback Loop for B Cell Response in Sepsis. Med Sci Monit. 2015;21:1548-55.

67. Goodwin AJ, Guo C, Cook JA, Wolf B, Halushka PV, Fan H. Plasma levels of microRNA are altered with the development of shock in human sepsis: an observational study. Crit Care. 2015;19:440.

68. How CK, Hou SK, Shih HC, Huang MS, Chiou SH, Lee CH, et al. Expression profile of MicroRNAs in gram-negative bacterial sepsis. Shock. 2015;43(2):121-7.

69. Liu J, Shi K, Chen M, Xu L, Hong J, Hu B, et al. Elevated miR-155 expression induces immunosuppression via CD39(+) regulatory T-cells in sepsis patient. Int J Infect Dis. 2015;40:135-41.

70. Mohnle P, Schutz SV, van der Heide V, Hubner M, Luchting B, Sedlbauer J, et al. MicroRNA-146a controls Th1-cell differentiation of human CD4+ T lymphocytes by targeting PRKCepsilon. Eur J Immunol. 2015;45(1):260-72.

71. Zhou J, Chaudhry H, Zhong Y, Ali MM, Perkins LA, Owens WB, et al. Dysregulation in microRNA expression in peripheral blood mononuclear cells of sepsis patients is associated with immunopathology. Cytokine. 2015;71(1):89-100.

72. Chen J, Jiang S, Cao Y, Yang Y. Altered miRNAs expression profiles and modulation of immune response genes and proteins during neonatal sepsis. J Clin Immunol. 2014;34(3):340-8.

73. Tacke F, Roderburg C, Benz F, Cardenas DV, Luedde M, Hippe HJ, et al. Levels of circulating miR-133a are elevated in sepsis and predict mortality in critically ill patients. Crit Care Med. 2014;42(5):1096-104.

74. Tudor S, Giza DE, Lin HY, Fabris L, Yoshiaki K, D'Abundo L, et al. Cellular and Kaposi's sarcoma-associated herpes virus microRNAs in sepsis and surgical trauma. Cell Death Dis. 2014;5:e1559.

75. Wang H, Yu B, Deng J, Jin Y, Xie L. Serum miR-122 correlates with short-term mortality in sepsis patients. Crit Care. 2014;18(6):704.

76. Wang L, Wang HC, Chen C, Zeng J, Wang Q, Zheng L, et al. Differential expression of plasma miR-146a in sepsis patients compared with non-sepsis-SIRS patients. Exp Ther Med. 2013;5(4):1101-4.

77. Wang HJ, Zhang PJ, Chen WJ, Jie D, Dan F, Jia YH, et al. Characterization and Identification of novel serum microRNAs in sepsis patients with different outcomes. Shock. 2013;39(6):480-7.

78. Shao Y, Li J, Cai Y, Xie Y, Ma G, Li Y, et al. The functional polymorphisms of miR-146a are associated with susceptibility to severe sepsis in the Chinese population. Mediators Inflamm. 2014;2014:916202.

79. Benz F, Roderburg C, Vargas Cardenas D, Vucur M, Gautheron J, Koch A, et al. U6 is unsuitable for normalization of serum miRNA levels in patients with sepsis or liver fibrosis. Exp Mol Med. 2013;45:e42.

80. Li Y, Dalli J, Chiang N, Baron RM, Quintana C, Serhan CN. Plasticity of leukocytic exudates in resolving acute inflammation is regulated by MicroRNA and proresolving mediators. Immunity. 2013;39(5):885-98.

81. Ma Y, Vilanova D, Atalar K, Delfour O, Edgeworth J, Ostermann M, et al. Genome-wide sequencing of cellular microRNAs identifies a combinatorial expression signature diagnostic of sepsis. PLoS One. 2013;8(10):e75918.

82. Roderburg C, Luedde M, Vargas Cardenas D, Vucur M, Scholten D, Frey N, et al. Circulating microRNA-150 serum levels predict survival in patients with critical illness and sepsis. PLoS One. 2013;8(1):e54612.

83. Wang H, Meng K, Chen W, Feng D, Jia Y, Xie L. Serum miR-574-5p: a prognostic predictor of sepsis patients. Shock. 2012;37(3):263-7.

84. Precone V, Stornaiuolo G, Amato A, Brancaccio G, Nardiello S, Gaeta GB. Different changes in mitochondrial apoptotic pathway in lymphocytes and granulocytes in cirrhotic patients with sepsis. Liver Int. 2013;33(6):834-42.

85. Sun X, Icli B, Wara AK, Belkin N, He S, Kobzik L, et al. MicroRNA-181b regulates NF-kappaB-mediated vascular inflammation. J Clin Invest. 2012;122(6):1973-90.

86. Wang H, Zhang P, Chen W, Feng D, Jia Y, Xie LX. Evidence for serum miR-15a and miR-16 levels as biomarkers that distinguish sepsis from systemic inflammatory response syndrome in human subjects. Clin Chem Lab Med. 2012;50(8):1423-8.

87. Wang HJ, Zhang PJ, Chen WJ, Feng D, Jia YH, Xie LX. Four serum microRNAs identified as diagnostic biomarkers of sepsis. J Trauma Acute Care Surg. 2012;73(4):850-4.

88. Wang H, Zhang P, Chen W, Feng D, Jia Y, Xie L. Serum microRNA signatures identified by Solexa sequencing predict sepsis patients' mortality: a prospective observational study. PLoS One. 2012;7(6):e38885.

89. Wang JF, Yu ML, Yu G, Bian JJ, Deng XM, Wan XJ, et al. Serum miR-146a and miR-223 as potential new biomarkers for sepsis. Biochem Biophys Res Commun. 2010;394(1):184-8.

90. Schmidt WM, Spiel AO, Jilma B, Wolzt M, Muller M. In vivo profile of the human leukocyte microRNA response to endotoxemia. Biochem Biophys Res Commun. 2009;380(3):437-41.

91. Vasilescu C, Rossi S, Shimizu M, Tudor S, Veronese A, Ferracin M, et al. MicroRNA fingerprints identify miR-150 as a plasma prognostic marker in patients with sepsis. PLoS One. 2009;4(10):e7405.
